# Targeting protein–protein interactions in the DNA damage response pathways for cancer chemotherapy

**DOI:** 10.1039/d1cb00101a

**Published:** 2021-06-21

**Authors:** Kerry Silva McPherson, Dmitry M. Korzhnev

**Affiliations:** Department of Molecular Biology and Biophysics, University of Connecticut Health Center Farmington CT 06030 USA korzhniev@uchc.edu +1 860 679 3408 +1 860 679 2849

## Abstract

Cellular DNA damage response (DDR) is an extensive signaling network that orchestrates DNA damage recognition, repair and avoidance, cell cycle progression and cell death. DDR alteration is a hallmark of cancer, with the deficiency in one DDR capability often compensated by a dependency on alternative pathways endowing cancer cells with survival and growth advantage. Targeting these DDR pathways has provided multiple opportunities for the development of cancer therapies. Traditional drug discovery has mainly focused on catalytic inhibitors that block enzyme active sites, which limits the number of potential drug targets within the DDR pathways. This review article describes the emerging approach to the development of cancer therapeutics targeting essential protein–protein interactions (PPIs) in the DDR network. The overall strategy for the structure-based design of small molecule PPI inhibitors is discussed, followed by an overview of the major DNA damage sensing, DNA repair, and DNA damage tolerance pathways with a specific focus on PPI targets for anti-cancer drug design. The existing small molecule inhibitors of DDR PPIs are summarized that selectively kill cancer cells and/or sensitize cancers to front-line genotoxic therapies, and a range of new PPI targets are proposed that may lead to the development of novel chemotherapeutics.

## Abstract 2

Targeting protein–protein interactions within the DNA damage response (DDR) pathways with small molecule inhibitors is reviewed here as a strategy to design novel cancer chemotherapeutics.

## Graphical abstract

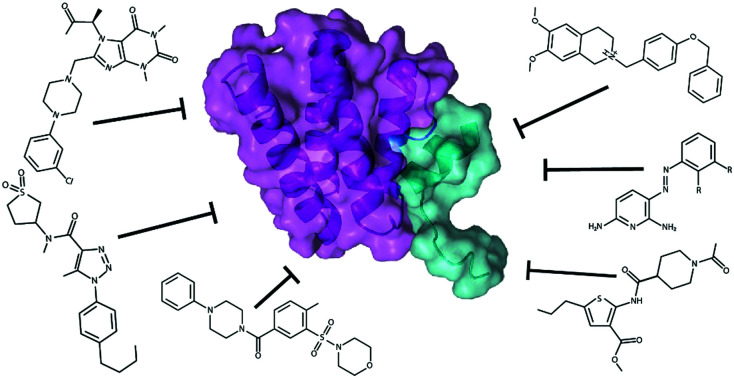

## 1. Introduction

The DNA damage response (DDR) is an extensive signaling network that combines cellular pathways collectively responsible for detection and recognition of DNA damage, DNA remodeling and repair, DNA damage bypass during replication, cell cycle control and cell fate decisions in response to DNA alternations.^1–5^ This network includes the ever-growing list of over 450 genes encoding proteins that orchestrate the appropriate response to numerous forms of DNA damage. The DDR is redundant and tightly coordinated both in time and in space with multiple factors responsible for the choice of DNA repair mechanism and crosstalk between the pathways. An appropriate and timely DDR is critical for maintaining genomic integrity and accurate passage of genetic information to the next generation of cells. Dysregulation and mutation of DDR genes may cause genome instability, uncontrolled cell growth, and avoidance of cell death, all of which are hallmarks of cancer.^2–7^

The DDR pathways and their therapeutic targeting have been subject to extensive investigation and review.^1–13^ DNA damage caused by various genotoxic agents elicits a complex response, which is contingent on the type of DNA lesion and the cell cycle status.^4,14^ The DDR is initiated by sensor proteins recognizing specific DNA substrates, which alert DDR kinases to transduce the signal. The master DDR kinases belong to the phosphatidylinositol 3-kinase-related kinase (PIKK) family and include DNA protein kinase (DNA-PK), ataxia telangiectasia and Rad3-related (ATR), and ataxia telangiectasia mutated (ATM).^15^ These kinases coordinate the DDR by phosphorylating substrates such as checkpoint kinases, DNA repair proteins, and apoptotic regulators. Checkpoint kinases are activated to freeze the cell cycle to allow time for DNA repair, while apoptosis or senescence can be initiated if the DNA damage is too detrimental for repair. The downstream DNA repair pathways are subsequently employed to reverse DNA damage, including double-stranded break (DSB) repair *via* non-homologous end-joining (NHEJ) or homologous recombination (HR), nucleotide excision repair (NER), base excision repair (BER), mismatch repair (MMR), and interstrand cross-link (ICL) repair through the Fanconi Anemia (FA) pathway.^3–5^ In addition to repair pathways, the DDR also utilizes DNA damage tolerance (DDT) pathways such as translesion synthesis (TLS), which allows bypass replication over DNA lesions at the price of increased mutagenesis.^16–19^

Alteration or loss of DDR capabilities is a hallmark of many types of cancer, which is often compensated by the use of alternative DDR pathways that can be exploited pharmaceutically to selectively kill cancer cells.^4–13^ Furthermore, genotoxic front-line chemotherapeutics induce DNA damage that can be repaired and/or avoided by specific DDR pathway(s), which can be targeted for the development of adjuvant drugs to enhance efficacy of existing treatments.^12,13,20^ Numerous DDR proteins have been proposed as chemotherapeutic targets, some of which have been inhibited successfully by drugs undergoing clinical testing or that have already received approval.^8–11^

The majority of drug discovery has traditionally focused on proteins that are deemed “druggable”, including enzymes (kinases, proteases, phosphodiesterases), ion channels, and G-protein coupled receptors,^21^ while protein–protein interactions (PPIs) were long considered “undruggable” due to their large, flat, and mostly hydrophobic interfaces.^22–29^ Typically, “druggable” proteins feature active sites within deep cavities with surface areas of 300 to 500 Å^2^ capable of binding soluble small molecule inhibitors. As of 2015, about 30% of approved anti-cancer drugs targeted protein kinases, which represent only an estimated 4% of DDR proteins.^6^ However, catalytic inhibition might not be the optimal strategy for drug development targeting DDR pathways,^12^ as catalytic inhibitors often have poor selectivity that may result in deleterious side effects.^25,26^ For example, DNA polymerases share similar active sites, making the design of inhibitors with high selectivity challenging.^13^ Furthermore, many proteins within DDR pathways do not have catalytic function and play roles in cellular localization, complex formation, and substrate recognition.^6^ To circumvent this issue and expand the range of drug targets, researchers are turning towards the development of a new class of therapeutics, inhibitors of DDR PPIs.^12,13^ Thus, clinically relevant PPI inhibitors such as tirofiban, an integrin disrupter to treat stroke patients,^24,30^ and Mdm2–p53 PPI inhibitors to treat cancer,^31,32^ spark optimism that disrupting PPIs is a promising avenue for drug design.

In this review, we will outline the process of structure-based design of small molecule PPI inhibitors and discuss the rationale for targeting PPIs in the DDR pathways for cancer therapy. Then, we will describe how disrupting various PPIs that mediate assembly of multi-protein DDR complexes, facilitate recruitment and activation of DDR factors, control DNA repair and damage tolerance, regulate cell cycle, and decide cell fate can pave the way for the development of novel cancer treatments. We will overview recent progress and provide specific examples of targeting the DDR network for anti-cancer drug design, as well as highlight potential new avenues for PPI inhibition within the DDR pathways that may lead to the development of novel chemotherapies.

## 2. Structure based design of PPI inhibitors

In contrast to forward pharmacology, where potential drugs are tested in cells or *in vivo*, and afterwards the therapeutic mechanism is investigated,^33^ targeting protein–protein interactions (PPIs) with small molecules generally requires a structure based drug design (SBDD) approach, a combination of structural and computational biology to design drugs with high specificity for a target molecule.^34–36^ While structure determination of protein complexes may be demanding, the SBDD approach benefits from extensive structural data already available in the Protein Data Bank (PDB)^37,38^ and robust computational methods for modeling structures of homologous proteins.^39,40^ As of 2020, the Protein Data Bank included 167 518 structures primarily solved by X-ray crystallography and nuclear magnetic resonance (NMR) spectroscopy, a 90 078 increase in the last decade. Additionally, the advancements in cryo-EM technology now enables structure determination of protein complexes above 64 kDa at resolution as low as 1.8 Å,^41–43^ substantially increasing the number of macromolecular structure depositions in the PDB. Ideally, SBDD for a PPI would utilize the structure of a protein in complex with its binding partner. However, if such a structure is unavailable, molecular docking driven by experimental restraints can be used to produce a computational model.^44,45^ SBDD targeting a PPI is also possible using an apo-protein structure, albeit less optimal.^25^

**Fig. 1:**
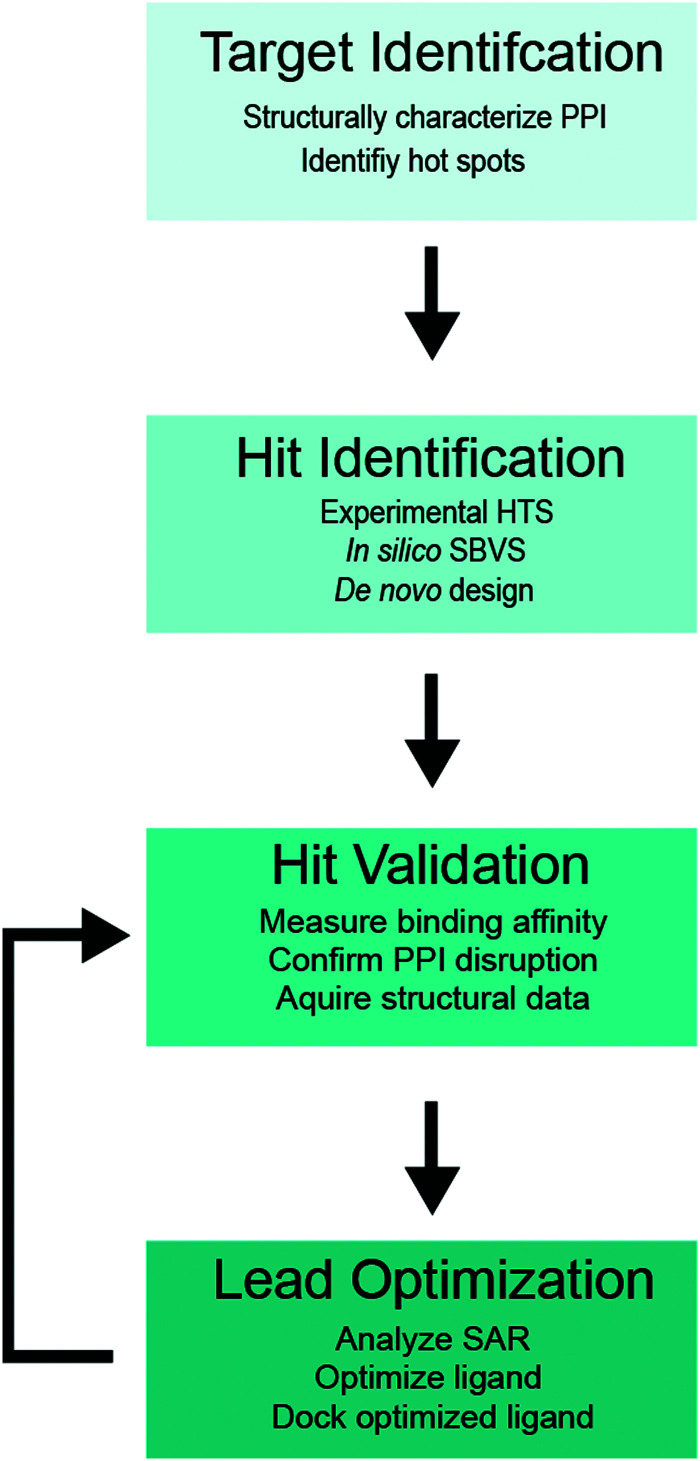
SBDD flowchart for PPI inhibitors.

A flowchart of the SBDD protocol for PPI inhibitors is shown in Fig. 1,^34–36^ which includes (i) structure determination of a protein complex and identification of potential target site(s) within the PPI interface (target identification), (ii) biochemical high-throughput screening (HTS)^46,47^ and structure-based virtual screening (SBVS)^48,49^ for candidate small molecule inhibitors (hit identification), (iii) experimental validation of small molecule binding to the target protein, PPI disruption, and structural characterization of small-molecule protein interaction (hit validation), and (iv) optimization of validated hits using computational, structural and binding studies to improve the ligand potency (lead optimization). Optimization is an iterative process, which requires multiple rounds of hit validation and lead optimization until desirable binding and pharmacokinetics properties are achieved.

### 2.1. Target identification

PPI interfaces usually cover 1000–3000 Å^2^ of protein surface area, which is much greater than the footprint of a typical drug-like small molecule, and on average 28 residues participate in binding.^24,50^ However, in most cases only a handful of critical residues within the PPI interface are responsible for the lion's share of binding free energy, appropriately termed “hot spots”. A strategy for PPI inhibition generally involves targeting these “hot spots” rather than blocking the entity of the interaction interface.^23–25,50–52^ Structural analysis of the PPI interfaces and identification of “hot spot” residues are the first steps in the SBDD protocol for a PPI of interest (Fig. 1). PPI interfaces often include core and rim areas, as well as support amino acids that are partially exposed in the apo-protein and become buried after binding. The core residues that create topological features on the PPI interface such as hydrophobic cavities or pockets are the primary sites for targeting with small molecules, while polar or charged rim residues that outline the core region, or support residues that fortify the PPI but contribute little binding free energy are less likely to participate in a “hot spot”.^50,51^ Since PPI “hot spots” are generally more hydrophobic than catalytic sites, inhibitors targeting PPIs also tend to be hydrophobic. Due to their decreased cellular uptake and solubility, hydrophobic ligands have smaller bioavailability, which should be considered throughout the design process.^24,25,52^

A combination of biochemical, structural and computational data can be used to identify “hot spots” on the PPI interface. Mutational analyses in immunoprecipitation, yeast 2-hybrid (Y2H) assays, and other binding studies can indicate potential “hot spot” residues, although these data should be interpreted with caution, as mutations may disrupt protein structure and/or alter dynamics.^25,52^ Therefore, structural data on a protein complex is invaluable for determining “hot spot” residues and cavities within the PPI interface that can be targeted with small molecules. Analyzing the interface topography and measuring the size of cavities on the PPI interface is plausible based on a static structure using software such as CAST.^53^ However, the detailed studies of binding energetics should also take into account the plasticity of the PPI interface, as proteins often undergo conformational changes upon binding consistent with an induced fit rather than a lock and key mechanism. Molecular dynamics (MD) simulations provide a powerful computational tool for “hot spot” identification, which enables calculation of binding energy contributions for individual residues within the PPI interface.^25,54–56^ MD simulations can also aid in identifying cavities of an appropriate size (250–900 Å^2^) to bind small molecules, as illustrated by the successful development of inhibitors targeting a hidden cavity in HIV integrase revealed by MD.^57^

An important consideration for choosing the PPI target is the demonstrated ability of PPI inhibition to abolish the function of the protein and/or cellular pathway (*i.e.* functional druggability of PPI), which can be established through cellular or *in vivo* studies.^12^ The loss of protein function can be validated in cellular assays by silencing the corresponding gene using siRNA or gene editing, while functional druggability of a PPI can be demonstrated through the inability of the PPI-disrupting mutants to functionally complement a knockout or knockdown gene in cellular and/or *in vivo* models. This functional target validation is usually performed in independent studies carried out before the intended design of PPI inhibitors.

### 2.2. Hit identification

Once the PPI target is selected, compound hits can be identified by biochemical high throughput screening (HTS)^46,47^ or *in silico* structure-based virtual screening (SBVS)^48,49^ of extensive libraries of drug-like compounds (Fig. 1). Alternatively, fragment-based drug design (FBDD) methods offer an advantage of probing large chemical space with a limited number of small (<200 Da) organic molecules to identify hit fragments weakly interacting with the target protein, which can be further combined and assembled into more potent compounds with drug-like characteristics.^58–60^

HTS is an established technology for identification of new lead compounds for drug development.^46,47^ A variety of experimental methods, including NMR spectroscopy,^61–65^ isothermal titration calorimetry (ITC),^66,67^ surface plasmon resonance (SPR),^68^ fluorescence polarization (FP) and intensity (FI) measurements,^69,70^ Förster resonance energy transfer (FRET),^71–73^ yeast 2-hybrid (Y2H) assays,^74–76^ microscale thermophoresis (MST),^77,78^ and others can be used to probe direct binding of compounds to a target protein and/or displacement of binding partner from a protein complex. Displacement assays provide a direct measure of PPI disruption by compounds and are preferable in HTS for PPI inhibitors.

Many of the above methods such as FP, Y2H and FRET have been successfully implemented in HTS format to identify small molecule inhibitors of PPIs.^69,72,76^ For example, measurement of FP changes for a small molecule fluorophore upon binding to a macromolecule or for a fluorescently tagged peptide upon displacement from a protein–peptide complex by an inhibitor has become a common method in HTS due to its sensitivity and low protein concentration requirements.^69,70^ Y2H assays performed in specialized yeast strains are advantageous for HTS because they select small molecules not only for PPI disruption but also for bioavailability. However, Y2H assays may suffer from low membrane permeability of inhibitors.^74–76^ Another favored method for HTS is FRET, which measures distance dependent fluorescence energy transfer between donor and acceptor fluorophores attached to interacting proteins and can be used for displacement assays *in vitro* or in cells.^71–73^ FRET is often chosen for HTS because *in vitro* assays require small amounts of protein,^71^ while cellular assays may be performed in physiologically relevant cell types.^72^ However, similar to FP, a disadvantage of FRET-based HTS is the use of protein tags that may affect protein binding, cellular localization, or expression levels.

SBVS is a powerful computational approach that enables rapid screening of virtual libraries of millions of drug-like compounds through their docking to a target site on a protein with known three-dimensional structure.^48,49^ SBVS can be performed using software packages such as DOCK,^79^ FlexX,^80^ SEED,^81^ Glide,^82^ GOLD^83^ and AutoDock^84^ by docking each small molecule in a library of virtual compounds to a target site on a protein to determine optimal position(s) and binding pose(s) of the compounds, and then sorting the compounds according to binding energy scoring function to select best hits.^56,60^ These *in silico* screening protocols are robust and cost effective, but have a tendency to yield false positives, which necessitates synthesis and biochemical evaluation of the best hits to test their ability to bind the target protein and disrupt the PPI.

Alternative to HTS and SBVS of full-sized drug-like compounds, which may result in very few hits, is FBDD that uses *in silico* or experimental screens to identify small organic molecules (<200 Da) with weak (high μM to mM range) binding affinities to a target protein.^58–60^ Following identification of such fragments, the hits are experimentally and computationally validated, and combined piece by piece into *de novo* designed drug-like compounds with increased binding affinity. While some of the methods for studying protein–ligand interactions are not suitable to probe sub-mM binding, the method of choice for fragment screening in the FBDD approach is ligand-based NMR, which may also be used during the subsequent validation step of SBDD.^61,62^

### 2.3. Hit validation

To eliminate false positives from HTS and SBVS screens and identify key structure–activity relationships (SAR) necessary for further development of the identified scaffolds as drug leads, the SBDD protocol usually includes a thorough hit validation step (Fig. 1).^34–36^ This step generally includes verification of the compound binding to the target protein and measurements of binding affinity, confirming disruption of a target PPI, precise mapping of the compound binding site on a target protein, and high-resolution structure determination of a protein in complex with the identified compound.

Protein–ligand binding studies and displacement assays to show PPI disruption by the identified small molecules can be performed with experimental techniques described in the previous section. However, preferred for binding studies are the quantitative methods that can demonstrate direct binding of the hit compound to the protein and measure affinity of this interaction, including NMR,^61–65^ ITC,^66,67^ SPR^68^ and MST.^77,78^ Provided that dissociation constant (*K*_d_) for the PPI is known, the binding affinity of the PPI inhibitor can be estimated from the concentration dependent displacement data (*e.g.* FP^69,70^ or FRET^71–73^) *via* the exact formula or the Cheng–Prusoff equation.^85,86^ Each method for validation of protein-compound binding presents advantages and challenges. For example, ITC measures heat released during titration of a ligand into protein (or *vice versa*) and determines not only the binding affinity, but also the binding enthalpy and stoichiometry.^66,67^ The protein is neither immobilized nor labeled, however, solubility is often a limitation to this technique. SPR can measure the binding affinity and binding kinetics for a ligand flown over the protein immobilized on a chip surface, and utilizes less protein than ITC, mitigating the solubility limitation.^68^ However, SPR requires immobilization of a protein which may affect binding. MST can measure the binding affinity by monitoring diffusion of a fluorescently tagged protein at various concentrations of added ligand in a temperature gradient created by an infrared laser in a capillary.^77^ MST has gained popularity because it requires small amounts of protein (pm–nm concentrations) and immobilization of the protein is not required. The downside of MST is the necessity to fluorescently label the protein, although the label-free MST has been proposed utilizing intrinsic fluorescence of protein aromatic residues.^78^

NMR spectroscopy is a versatile method commonly used throughout the drug design process, which enables high-resolution mapping of the protein–ligand interface and accurate measurements of the binding affinity.^61–65^ This information is usually derived from protein-based NMR titration series,^61–63^ in which the ligand is gradually added to solution of an isotopically (^15^N and/or ^13^C) labeled protein, monitored by ^1^H–^15^N or ^1^H–^13^C correlation spectra recorded at each step of the titration. The ligand binding induces NMR chemical shift changes for protein residues in the vicinity of the binding site, providing accurate mapping of the binding interface, while protein chemical shifts measured *vs* ligand concentration allow extraction of dissociation constant (*K*_d_). Protein-based NMR can detect weak protein–ligand interactions with affinities in high μM to mM range, and, therefore, is a suitable choice for identification of fragments that weakly bind to a protein at early stages of FBDD. Limitations of protein-based NMR include the necessity for protein isotopic labeling, NMR resonance assignments, and high concentrations (>50 μM).^61–63^ Conversely, protein–ligand interactions can be studied by ligand-based NMR wherein an unlabeled protein is added into a small molecule solution monitored by 1D ^1^H or ^19^F spectra of the ligand, which can pinpoint atoms within the ligand that interact with the protein.^61–65^ Common techniques in ligand-based NMR include Water–ligand observed *via* gradient spectroscopy (WaterLOGSY)^61–64^ and saturation transfer difference (STD)^61–63,65^ experiments, which exploit nuclear Overhauser effect (NOE) magnetization transfer between the protein or transiently bound water and the ligand and can detect binding with *K*_d_ in the μM–mM range. Since ligand-based NMR requires little protein (0.5–50 μM), it is also practical to utilize this technique for HTS hit identification.^61,62^

Structure determination of protein–small molecule complexes by X-ray crystallography is an integral step in the SBDD hit validation commonly used to verify ligand binding to the target interface and identify critical SAR for hit compounds.^87,88^ Crystallization of a ligand–protein complex is usually performed by crystal soaking whereby an inhibitor is incubated with protein crystals, or co-crystallization whereby an inhibitor is mixed with protein solution prior to crystallization. Each of these methods has advantages and limitations. During soaking, the ligand might perturb the crystal and adulterate diffraction or fail to bind to the interface buried within the crystal lattice, while during co-crystallization, the protein–ligand mixture might not crystallize in the same conditions as the apo-protein, so that a new crystallization buffer may need to be identified.^87^ If diffracting crystals are obtained, extra caution must be taken during the structure refinement to establish the presence of a ligand, as crystallization protocols often result in apo-protein crystals. Furthermore, even when the ligand is present, partial occupancy and flexibility may contribute to ambiguity in the electron density. Therefore, researchers should always check that the SAR is chemically reasonable, as steric clashes or lack of contacts that contribute to binding might indicate an error in ligand modeling.^87,88^ Recently, structure determination by cryo-EM has gained popularity to identify SAR for ligand protein interactions.^41,42,89^ Cryo-EM requires less protein than X-ray crystallography and may be preferred if the protein is very large or a cryo-EM structure already exists.

### 2.4. Lead optimization

Structural and binding data acquired during the validation step of SBDD are subsequently used to optimize the identified hit compounds to become the inhibitor leads (Fig. 1). The goal of this optimization is to utilize the available SAR to introduce modifications in the identified small molecule scaffolds to improve potency and/or binding affinity, while also addressing potential pharmacokinetic (PK) liabilities.^34–36^ To optimize binding properties, chemical groups may be added to facilitate favorable interactions with the protein, or removed if unfavorable contacts or clashes are observed. Docking protocols and MD can be used to predict contributions to binding energy for individual groups of the original and modified ligand, as well as probe flexibility of the protein and inhibitor.^56,60^ Rigidity is an important factor to be considered during optimization, as rigid molecules tend to exhibit favorable affinities due to less entropy lost upon binding.^90^ During optimization, multiple factors contributing to drug-like properties such as solubility, membrane permeability, metabolic stability and others should be considered to design compounds with favorable physicochemical and absorption, distribution, metabolism, excretion and toxicity (ADMET) profiles.^91,92^ As noted previously, optimization is an iterative process. Once changes are made to the initial hit, the new molecules should be validated and the SAR reassessed.

## 3. Targeting the DDR for cancer treatment

The DDR is frequently altered in cancers by either genetic mutation or aberrant expression of DDR genes.^2–7^ A dysregulated DDR enables unregulated growth of cancer cells, increased viability during stress, avoidance of cell death, and genome instability. A deficiency in a specific DDR pathway is often compensated by a dependency on an alternative pathway essential for cancer cell survival, which provides therapeutic opportunity to selectively kill cancer cells.^4–13^ Therefore, targeting DDR pathways has become a popular strategy for the development of anti-cancer therapy.^8–13^ This section will introduce the general modes of killing cancer cells *via* DDR inhibition, while the following sections will overview the major DDR pathways and discuss current and potential pharmaceutical targets within the DDR network with the specific focus on druggable PPIs.

### 3.1. Synthetic lethality

Synthetic lethality is a phenomenon whereby two genes are simultaneously disrupted to induce cell death, while mutation of only one gene does not.^93,94^ Cancer cells that have lost the function of one DDR gene may rely on another gene for survival. By identifying a deficient gene, a second gene can be pharmaceutically inhibited to cause a synthetic lethal phenotype, thereby selectively killing cancer cells that harbor a mutation. This concept has been successfully applied to develop poly(ADP-ribose)polymerase (PARP) inhibitors (PARPi) that specifically target BRCA deficient cancers with defective HR.^95–98^ PARPs are a family of proteins that recognize specific types of DNA damage best known for their roles in DNA single-strand break repair (SSBR).^4,99–101^ PARP inhibition causes accumulation of SSBs and their replication-dependent conversion to deleterious double-strand breaks (DSBs) that require functional HR for repair. The HR deficient cells are unable to efficiently cope with excessive DSBs during S-phase leading to cell death. The clinical approval of PARP inhibitors such as Niraprib, Olaparib, Talazorparib, and Rucaparib as monotherapies for BRCA deficient breast and ovarian cancers provides strong evidence that inducing synthetic lethality is a powerful approach for killing cancer cells with robust specificity.^9^

### 3.2. Sensitization to DNA damaging agents and averting resistance

The mainstream treatment for the majority of cancers remains to be the use of genotoxic agents that induce DNA damage.^102–106^ The elevated levels of DNA damage and replication stress characteristic of cancer cells can be further enhanced by genotoxic chemotherapies such as platinating and alkylating agents, causing replication catastrophe, induction of apoptosis and cell death.^102–106^ Cisplatin and other platinating agents covalently bind DNA bases, causing inter- and intra-strand crosslinks,^102–105^ while alkylating agents such as cyclophosphamide form bulky DNA adducts.^106^ These modifications impede replicative DNA polymerases, cause replication fork stalling and collapse, and trigger apoptosis or senescence. On the other hand, ionizing radiation (IR) and topoisomerase 2 inhibitors such as etoposide, camptothecin, and doxorubicin kill cancer cells by inducing DNA DSBs.^10,107,108^ DNA damage induced by genotoxic agents may be repaired and/or tolerated by various DDR pathways, which can be targeted to enhance efficacy of front-line therapies.^12,13,20^ Furthermore, resistance to these therapies can develop due to a hyperactive DDR, so that targeting DDR pathways can also restore chemosensitivity of drug-resistant cancers. For example, upregulation of ATM, ATR, DNA-PK, and DSB repair proteins may cause radio- or chemo-resistant phenotypes, which can be reversed by inhibiting the apical DDR kinases and DSB repair pathways to sensitize cells to topoisomerase inhibitors and radiotherapy.^20,109,110^ Resistance to platinating and alkylating agents may be facilitated by an increase in DNA damage repair pathways like NER and BER that repair the covalently modified DNA bases.^111,112^ Additionally, TLS can promote resistance to genotoxic chemotherapy due to its ability to avoid DNA damage during replication.^113–116^ TLS is extremely error-prone and can further the genomic instability of cancer cells by introducing mutations that contribute to chemo-resistant phenotypes.^16–19^ Therefore, TLS inhibition was proposed as a strategy to sensitize cancers to genotoxic chemotherapy and avert chemoresistance by preventing mutagenesis.^13,117,118^

### 3.3. Targeting cell cycle dependent pathways

Another motive for DDR inhibition is to target cell cycle dependent DDR pathways that promote rapid division of cancer cells. The DDR is active throughout the cell cycle, but some repair pathways are more active during S, G2 and M phases.^4,14^ Since uncontrolled growth is a hallmark of cancer, targeting pathways necessary for proliferation opens an avenue for selectively attacking cancer cells. Thus, TLS and HR are predominantly utilized during S-phase for the replicative bypass of DNA lesions and DSB repair, and can be inhibited for this mode of cytotoxicity.^10–13^ The apical kinase ATR mainly used in S-phase is another cell-cycle dependent chemotherapeutic target.^10,11,15^

### 3.4. Inducing cell death programs

Stabilizing apoptotic pathways will reverse cancer's ability to evade cell death. The tumor suppressor, p53, discussed in further detail later in this review, is a transcription factor that regulates transcription of genes involved in numerous processes, including cell cycle arrest and cell death *via* senescence and apoptosis.^119,120^ The importance of p53 for guarding cells against the malignant transformation cannot be overstated, as the TP53 gene encoding the tumor suppressor is by far the most frequently mutated gene in cancers.^119,120^ The cellular levels of p53 are normally kept low though its ubiquitination by the E3 ligase, Mdm2, which tags p53 for proteasomal degradation.^119–121^ However, under conditions of genotoxic stress, p53 is stabilized in the cell and its levels increase to trigger cell cycle arrest and DNA repair, or apoptosis and cell death. Considering the importance of p53 for cancer etiology, cellular stabilization of p53 and other apoptotic promoting factors has emerged as a promising strategy for selectively killing cancer cells or sensitizing cancers to existing chemotherapies.^32,122–127^

## 4. Inhibition of apical PIKK kinases

**Fig. 2:**
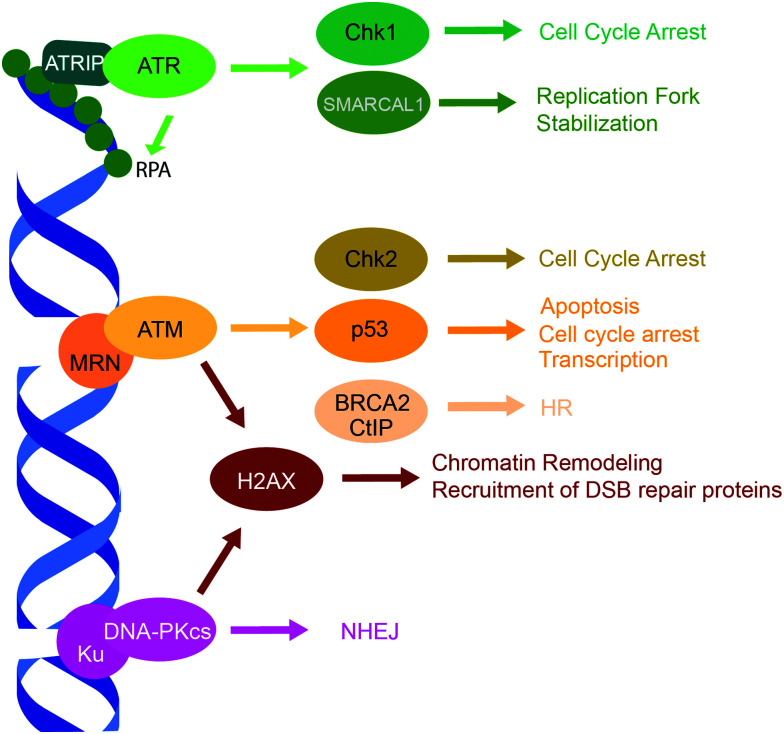
PIKK family DDR kinases, ATR, ATM, and DNA-PK, and their representative substrates in a DDR signaling cascade.

Three related protein kinases within the PIKK family, ATR, ATM and DNA-PK, are primarily responsible for orchestrating the appropriate response to DNA damage (Fig. 2).^15^ While each of the three kinases have unique phosphorylation substrates, they also share considerable functional redundancy.^15,128–130^ ATM, ATR, and the DNA-PK catalytic subunit (DNA-PKcs) have similar structural organization and are comprised of four structured domains, including HEAT repeats, a FRAP-ATM-TRRAP (FAT) domain, a PIKK regulatory domain, and a kinase domain.^15^**ATR** is a central kinase in the response to DNA replication stress, which is activated by ssDNA coated by the replication protein A (RPA) that binds and stabilizes ssDNA regions accumulated following DNA damage at stalled replication forks.^15^ The ATR-interacting protein (ATRIP) binds RPA, and recruits the ATR/ATRIP complex,^131,132^ while the activator proteins TopBP1 and ETAA1 bind ATR *via* their ATR-activation domains and stimulate its kinase activity to initiate DDR.^133–135^ ATR has hundreds of phosphorylation substrates, many that overlap with ATM. Substrates of note include checkpoint kinase 1 (Chk1), which facilitates cell cycle arrest, and RPA and SMARCAL1, which stabilize the replication fork.^15,128^**ATM** regulates the cellular response to DNA DSBs, and is recruited to DSB ends by the MRE11-Rad50-NBS1 (MRN) complex *via* a PPI between the N-terminus of NBS1 and ATM.^132,136,137^ ATM has hundreds of phosphorylation substrates such as histone H2AX, p53, checkpoint kinase 2 (Chk2), and DSB repair proteins, 53BP1 and BRCA1.^15,128,129^**DNA-PK** is a regulator of DSB repair by NHEJ, which is comprised of two subunits, the catalytic subunit DNA-PKcs and Ku. Ku is a heterodimer of Ku70 and Ku80 subunits that recognizes and binds DSB ends,^138,139^ thus recruiting and activating DNA-PKcs.^15,140^ DNA-PK has numerous phosphorylation substrates, including itself, H2AX, p53, and various NHEJ proteins.^15,130^

### 4.1. Pharmaceutical targeting of PIKK kinases

Targeting the master DDR PIKK family kinases provides multiple avenues for anti-cancer drug design. First, PIKK inhibition amplifies genotoxic chemo- and radio-therapy sensitivity.^11,15^ Second, synthetic lethal relationships exist between the PIKK family members and other proteins commonly affected in cancer, such as between ATM and ATR, ATM and DNA-PK, ATR and Chk1, and ATM and p53, which can be exploited for the development of selective cancer therapeutics.^11,15,141^ Additionally, uncontrolled growth of cancer cells is often dependent on ATR signaling, and inhibition of ATR was shown to be more cytotoxic to cancer cells than normal cells.^142^ Multiple inhibitors of the PIKK family have been designed and tested for their use as anti-cancer agents, and those undergoing clinical trials have been extensively reviewed by Cleary *et al.*^9^ and Brown *et al.*^11^ ATR, ATM, and DNA-PK inhibitors targeting the active site or the ATP-binding pocket often lack selectivity against a specific kinase.^9,11^ Furthermore, this mode of inhibition may result in accumulation of catalytic dead kinases at the chromatin, causing more genomic instability than catalytic inhibition itself. This steric hindrance of kinases at chromatin may result in the increased toxicity of PIKK inhibitors.^143,144^

### 4.2. PPI targets for PIKK inhibition

Disruption of PPIs that mediate recruitment and activation of the PIKK family kinases may provide a viable strategy for the development of selective and less toxic PIKK inhibitors. ATR is recruited to RPA-coated ssDNA through PPIs with ATRIP and activated through PPIs with partners such as TopBP1^15,131,132^ and ETAA1,^15,133–135^ while the MRN complex (MRE11-Rad50-NBS1) and the Ku70/Ku80 heterodimer recruit and activate ATM^15,132,136,137^ and DNA-PK,^15,140^ respectively. ATRIP, Ku80, and NBS1 have motifs in their C-terminus with a consensus sequence of EExXXXDDL (where ‘X’ is any residue, ‘x’ is any residue or a gap) that bind to HEAT repeats of ATR, DNA-PKcs, and ATM.^132^ Structural data is available to aid the SBDD for this PPI, albeit at low resolution.^145–151^ Two cryo-EM structures have been reported for the ATR–ATRIP complex in *H. sapiens* at 4.7 Å and *S. cerevisiae* (Mec1-Ddc2) at 3.9 Å (PDB: 5YZ0 and 5X6O),^145–147^ which may aid SBDD of inhibitors against this PPI. The PPI stoichiometry and interfaces for human and yeast ATR–ATRIP complexes are compared and discussed in Baretić *et al.*^147^ Two cryo-EM structures of the DNA-PK holoenzyme are available at 6.6 Å and 5.8 Å resolution,^148–150^ revealing that the Ku/DNA-PKcs complex contains interfaces between the M-HEAT repeat of DNA-PKcs and Ku70, the N-HEAT repeat of DNA-PKcs and the bridge region formed by Ku70/Ku80, and M-HEAT and the Ku80 C-terminal domain (Ku80-CTD). These structural studies also confirmed the previous finding that the Ku80-CTD is a DNA-PKcs binder.^152^ While no structure of the NBS1-ATM has been reported, a 4.7 Å cryo-EM structure revealed the homo-dimerization interface of ATM, which may provide insight into PPIs necessary for ATM activity (PDB: 5NP0).^147,151^ Overall, the PIKK PPIs may provide potential targets for anti-cancer drug design, however, the limited low-resolution structural data available at this time present challenges for developing highly specific small molecule inhibitors of these PPIs.

## 5. Targeting checkpoint kinases and cell fate regulators

Cell cycle arrest and cell death pathways such as apoptosis or senescence are coordinated by numerous downstream targets of the PIKK family DDR kinases (Fig. 2).^15,119,120,153^ Mutations associated with these proteins are prevalent in cancer and they have been extensively studied as chemotherapeutic targets.^9,154^ Although copious DDR proteins are involved in cell cycle stalling and determining cell fate, here we focus on the three key DDR regulators of cell fate and cell cycle, the tumor suppressor p53^119,120^ and the checkpoint kinases Chk1 and Chk2.^153^

### 5.1. Targeting p53–Mdm2

**Fig. 3:**
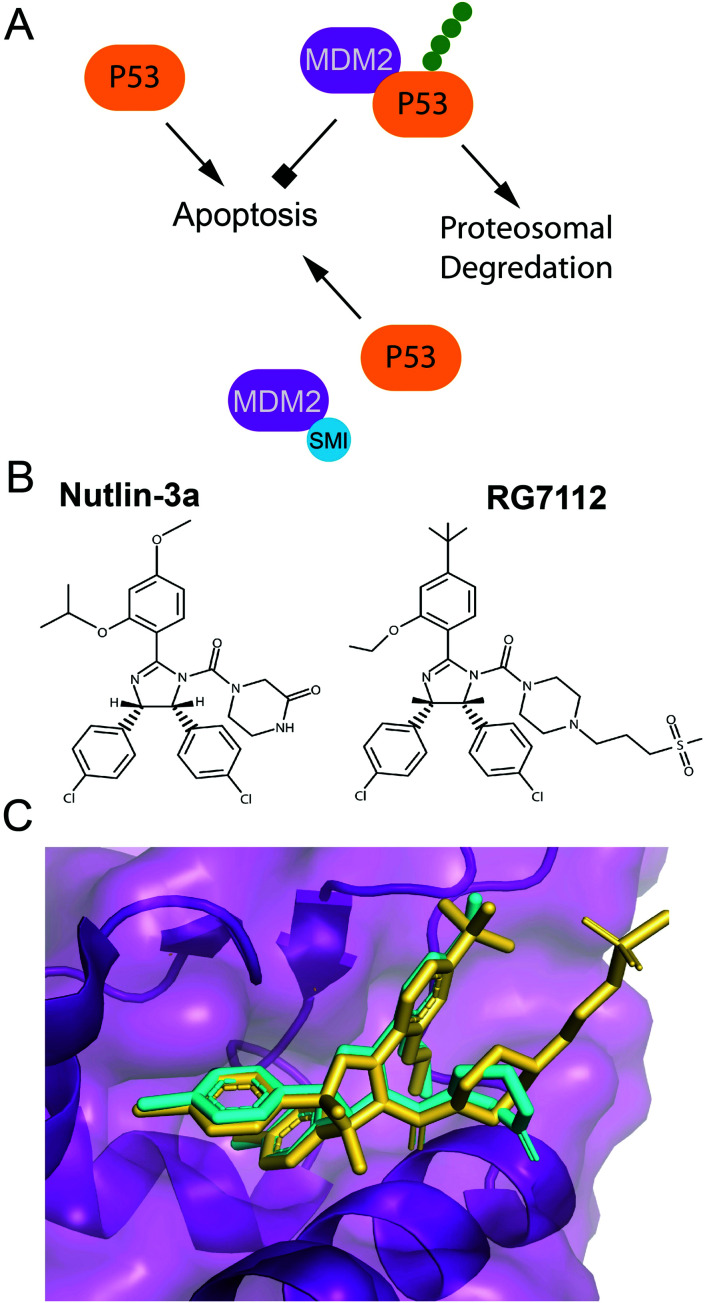
Pharmaceutical disruption of the Mdm2–p53 PPI. (A) Schematic of p53 stabilization by a small molecule inhibitor. (B) Nutlin-3 and RG7112 structures. (C) Nutlin-3 (cyan) (PDB: 4J3E) and **RG7112** (yellow) (PDB: 4IPF) in complex with the p53 binding site of Mdm2.

P53 is a tumor suppressor and transcription factor that transcriptionally regulates cellular responses to DNA damage, including cell cycle arrest, apoptosis and senescence.^119,120^ P53 levels in a cell are regulated by a ubiquitin ligase Mdm2 that tags p53 for proteasomal degradation.^119–121^ In response to DNA damage, p53 is stabilized either through its direct phosphorylation by ATM or other protein kinases, or through phosphorylation of Mdm2. These phosphorylation events disrupt the interaction of p53 and Mdm2, and prevent p53 ubiquitination and prosomal degradation (Fig. 3A). Therefore, a number of small molecule inhibitors of the Mdm2–p53 PPI that stabilize p53 and induce apoptosis have been developed as anticancer agents, several of which progressed to clinical trials (Fig. 3B).^32,122–125^ Additionally, several small molecule re-activators of p53 variants mutated in cancers have been developed and tested in clinical trials.^122,126^ Finally, an alternative strategy for p53 stabilization *via* inhibition of the p53 deubiquitinating enzyme, USP7, has been also explored.^127^

A 2.60 Å resolution crystal structure of the N-terminal domain of Mdm2 bound to the 15-residue transactivation domain (TAD) peptide of p53 (PDB: 1YCR) revealed that p53 residues F19, W23, and L26 bind inside a hydrophobic pocket on Mdm2.^155^ This structure was utilized in SBDD by several research groups to create potent p53 stabilizing inhibitors that disrupt the Mdm2–p53 PPI, which are reviewed extensively (Fig. 3B and C).^32,122–125^ Nutlins were the first group of Mdm2–p53 PPI inhibitors, which bind to Mdm2's hydrophobic pocket and mimic the FWL motif of p53.^31^ A nutlin analog, **RG7112**, was the first to be clinically tested but resulted in hematological toxicity.^156,157^ Seven Mdm2–p53 PPI inhibitors are currently in clinical trials.^124,125^

### 5.2. Chk1 and Chk2 inhibition

The downstream targets of ATR and ATM are serine/threonine checkpoint kinases, Chk1 and Chk2, that regulate the intra-S and G2/M checkpoints and the G1/S checkpoint, respectively, to initiate cell cycle arrest and DNA repair (Fig. 2).^15,153^ Like the PIKK family kinase inhibitors, early inhibitors of Chk1 and Chk2 had poor selectivity, while later inhibitors exhibited higher specificity. Over 70 Chk1-inhibitor structures have been reported, a few of which have progressed to clinical trials, reviewed in Dent *et al.*^158^ and Ronco *et al.*^154^ Fewer Chk2 inhibitors have been reported, and thus far, inhibition of Chk1 is favored as a chemotherapeutic strategy.^154^ Chk1 inhibition may sensitize BRCA deficient cells to PARPis,^159^ and Chk1 and ATR have a synthetic lethal relationship.^154^ However, a number clinical trials of Chk1 inhibitor were terminated due to toxicity, and no trials so far have progressed to phase III, suggesting the mechanism of Chk1 inhibition might warrant improvement.^158^

Similar to the PIKK family kinases, targeting Chk1 PPIs may provide a novel strategy to developing inhibitors with lower toxicity. For example, Chk1 activation and ATR–Chk1 interaction within a multi-protein complex formed on ssDNA are mediated by Chk1 PPIs with S/T-phosphorylated ExxxLC(S/T)GxFE repeats of an adaptor protein, Claspin.^160^ Small molecule inhibitors of this PPI would impede Chk1 activation, and since Claspin expression is elevated in many cancers, could have advantageous clinical applications.^161,162^ A recent 1.8 Å resolution X-ray crystal structure of Chk1 in complex with a high-affinity phosphorylated Claspin motif (PDB: 7AKO) provides a structural basis for the development of inhibitors against this PPI.^163^ Another PPI of interest is the Chk2 dimer interface. Chk2 phosphorylation by ATM induces Chk2 dimerization and promotes its autophosphorylation and activation of kinase activity.^164,165^ Li-Fraumeni syndrome, a predisposition to cancer, is caused by I157T mutation, which disrupts dimerization and autoactivation of Chk2.^164^ A 3.25 Å resolution dimeric crystal structure of Chk2 (PDB: 3I6W) reveals an expansive dimer interface with connections in the forkhead association (FHA) domain, the kinase domain, and the loop between FHA and kinase domains.^165^ I157T mutation abolished dimerization *in vitro*, identifying a hotspot on the dimer interface, while W97A mutation drastically reduced Chk2 dimerization, suggesting another potential hot spot that can be targeted with small molecule inhibitors.

## 6. Targeting DSB repair

### 6.1. DSB repair pathways

DSBs are extremely cytotoxic and, if unrepaired, can cause chromosomal translocations and cell death. Substrate recognition by sensor DNA binding proteins, including Ku and the MRN complex, and subsequent intracellular signaling initiates DSB repair by one of two major pathways, non-homologous end-joining (NHEJ) or homologous recombination (HR) (Fig. 2).^3–5,166–169^ The choice of DSB repair pathway is dictated by the extent of DNA end resection at the break.^166^ While classical NHEJ does not require extensive DNA end processing, HR is dependent on end resection to create ssDNA overhangs. Paramount for NHEJ is the protection of blunt ends and negative regulation of end resection, which is achieved through phosphorylation of p53-binding protein 1 (53BP1) by ATM followed by RAP1-interacting factor 1 (RIF1) dependent recruitment of the shieldin complex to DSB ends.^170–175^ The shieldin complex directly binds the CST complex, which recruits the Polα primase to remodel ssDNA ends into blunt ends needed for NHEJ.^170,171^ Proteins that promote NHEJ and HR generally act antagonistically to each other, with a reduction in NHEJ pathway choice promoting HR and *vice versa*.^166,176^ HR is commonly employed during S and G2-phases of the cell cycle and is a high-fidelity pathway, while NHEJ repairs the majority of DSBs throughout the cell cycle and is error-prone.^3–5,166–169^ The two secondary DSB repair pathways, alternative non-homologous end-joining (alt-NHEJ) and single-strand annealing (SSA), are error-prone and require DNA end resection.^166,167^

**Fig. 4:**
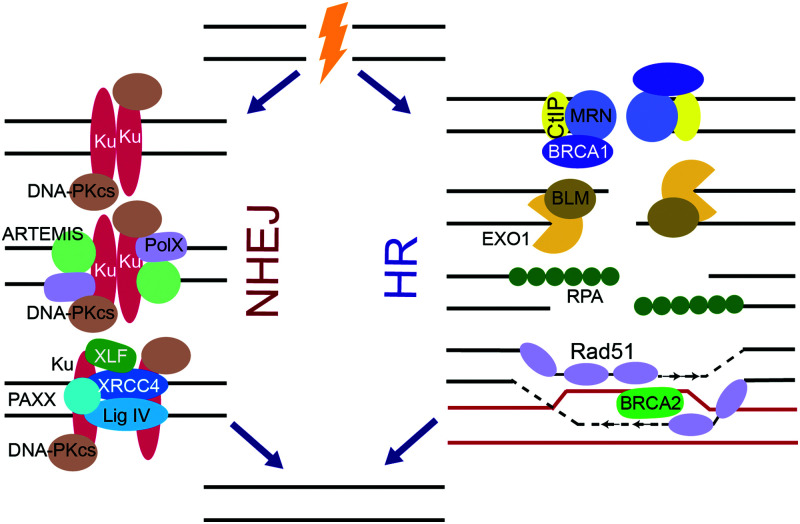
HR and NHEJ, the two major NHEJ pathways. For NHEJ, DSB ends are recognized by Ku, which binds and activates DNA-PKcs. DNA nucleases and X-family polymerases Pol λ or μ remodel DSB ends. The DSBs are ligated by the ligation complex containing Lig IV, XLF, PAXX, and XRCC4. For HR, ends are resected by the MRN/BRCA1/CtIP complex and then various nucleases, resulting in long stretches of RPA-coated ssDNA. RPA is replaced by Rad51 in a BRCA2 dependent manner, and Rad51 facilitates strand invasion of a homologous DNA sequence, which is used as a template for DNA synthesis. After synthesis, the resulting HR intermediates are resolved and the ends are ligated.

***NHEJ*** is very efficient in repairing DNA DSBs and plays the primary role in protecting genome integrity despite its error-prone nature (Fig. 4).^3–5,167,168^ DSB ends to be repaired by NHEJ are recognized by the Ku70/Ku80 heterodimer (Ku) that binds to the DSB ends and activates DNA-PKcs.^138,139^ Together, Ku and DNA-PKcs form the DNA-PK complex that binds, phosphorylates and activates various NHEJ proteins. Ku serves as a scaffold for the recruitment of proteins that remodel the DNA break ends such as ARTEMIS, a pivotal NHEJ nuclease, and X-family DNA polymerases λ and μ. X-Ray Repair Cross Complementing 4 (XRCC4), XRCC4-like factor (XLF), Paralog of XRCC4 and XLF (PAXX), and DNA ligase IV (Lig IV), in conjunction with Ku align and ligate the two DNA ends.

***HR*** provides a high-fidelity alternative to NHEJ during S and G2-phases, which can repair DNA DSBs in an error-free manner by using a sister chromatid as template (Fig. 4).^3–5,169^ When a DSB is to commit to HR repair pathway choice, the DSB end resection is controlled by ATM trough CtBP interacting protein (CtIP), which forms a multi-protein complex with MRN and BRCA1. The MRN complex, which is initially recruited to DSBs by PARP, resects the DSB ends to form long stretches of ssDNA. Next, Bloom syndrome helicase (BLM) unwinds the DNA ends, while exonuclease 1 (EXO1), the Sgs1-Top3-Rmi1 (STR) complex, and endonuclease DNA2 perform further resection to the DNA ends, forming large 3′ ssDNA overhangs. RPA bound to the overhangs is subsequentially displaced by the recombinase, Rad51,^177,178^ leading to assembly of Rad51 filaments on ssDNA mediated by BRCA2. Rad51 invades duplex DNA and seeks out a homologous sequence, usually a sister chromatid. Rad51 facilitates base pairing of the 3′ overhang with the template DNA, and the template DNA displaces from its complementary strand forming a displacement loop. Polδ replicates the template strand, extending the 3′ overhang. After replication, the newly synthesized 3′ end ligates to the 5′ end of the DSB, thus resolving the DSB.

***Alt-NHEJ and SSA*** are auxiliary homology-driven DSB repair mechanisms that require DNA end resection.^166,167,179–182^ Both pathways are extremely error prone and may cause gross genome rearrangements, and thus are implicated in oncogenesis. To join DNA ends, alt-NHEJ uses <25 bp microhomology, while SSA requires longer homology regions. In **alt-NHEJ**, the MRN complex accumulates at DSBs in a PARP1-dependent manner and resects DNA ends to form 15–100 bp ssDNA overhangs. DNA polymerase, Polθ, aids in remodeling of the DSB and is responsible for the mutagenic insertions caused by alt-NHEJ, while ligases I or III ligate the remodeled ends together. Alt-NHEJ is hyperactive in HR deficient cells and inhibition of Polθ is synthetic lethal with many HR related genes such as BRCA1 and BRCA2.^179–181^ Another mutagenic homology-driven DSB repair pathway, **SSA**, utilizes direct annealing of the resected ssDNA regions with homologous sequence by the Rad52 protein, with the resulting DNA flaps removed by XPF/ERCC1.^166,167,182^

### 6.2. NHEJ inhibition

#### *Ku PPIs and inhibition*

**Fig. 5:**
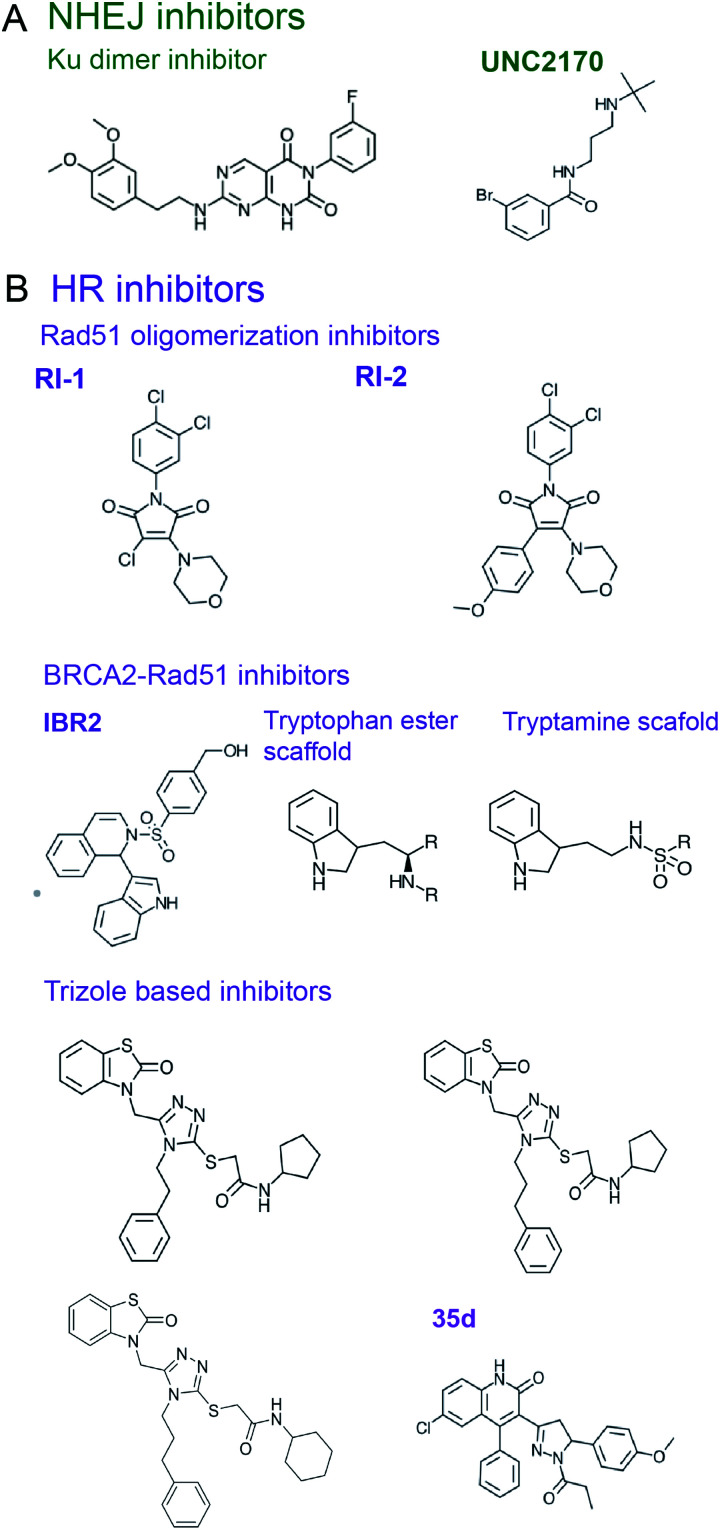
Inhibitors of DSB repair by (A) NHEJ and (B) HR.

Inhibition of PPIs mediated by the Ku complex,^138,139^ which recognizes and protects DSB ends and serves as a binding platform for NHEJ proteins, provides a promising strategy to attenuate NHEJ. Thus, Ku deficiency was shown to abrogate NHEJ in cells and promote both chemo- and radio-sensitivity.^183,184^ Early studies identified residues 449–477 of Ku80 as the minimal Ku70-binding region, and the double AV453,454HH mutation was shown to abrogate this PPI,^185^ which led to the suggestion that a peptide encompassing this region may be used as a probe to screen for Ku dimerization inhibitors.^12^ The 2.5 Å and 2.7 Å resolution X-ray crystal structures of Ku and its complex with DNA (PDB: 1JEQ, 1JEY) revealed the structural organization of a ring-shaped Ku70/Ku80 heterodimer and details of its interaction with DSB ends.^186^ SBDD and *in silico* SBVS were used to identify an inhibitor to bind a pocket within the dimer interface and the neighboring the DNA-interacting region formed by residues Y400, L256, and R486 in Ku70 and Q269, N359, and R486 in Ku80.^187^ Initially, nine compounds were identified using *in silico* screening to bind the pocket, one of which was experimentally validated to inhibit Ku–dsDNA binding and DNA-PKcs activation with an IC_50_ of 3.5 μM and 2.5 μM, respectively (Fig. 5A). Although docking of the compound into the Ku complex was performed, further biochemical and structural studies are necessary to verify its mechanism of action and optimize into a drug lead.

As a scaffold, Ku mediates various PPIs to recruit NHEJ proteins, which can be explored as potential targets for the development of small molecule NHEJ inhibitors. Thus, Ku binds PAXX, a protein that supports end ligation and is synthetic lethal with XLF, whose mutants confer radio- and chemo-sensitivity.^167,188^ PAXX's C-terminus (residues 177–204) binds Ku, and PAXX point mutants V199A and F201A disrupted the PAXX-Ku PPI and sensitized cells to IR treatment, identifying a PPI ‘hot spot’.^188^ Ku also binds the C-terminus of XLF, which forms a homodimer within the ligation complex. Deletion of the XLF C-terminal domain not only disrupts the XLF-Ku PPI, but also mitigates recruitment of XRCC4 to DSB ends, suggesting this PPI is crucial to the ligation complex.^189^ As seen in the crystal structures of Ku bound to an XLF peptide, the last four residues of XLF (GLFS) bind a hydrophobic pocket of Ku80 (PDB: 6ERH, 6ERG).^190^ Mutations of L297 in this Ku-binding motif (KBM) of XLF disrupted its localization to Ku and also decreased XLF-XRCC4 filament formation.

***XRCC4 PPIs*** necessary for NHEJ's ligase complex formation, which consists of XRCC4, XLF, PAXX and LigIV (or Lig4) and aids in tethering DSB ends, provide potential targets for NHEJ inhibitor design.^167^ Thus, XRCC4–Lig4 PPI is essential in DSB end ligation, with deficiency in either of the binding partners or deletion of the Lig4-binding region of XRCC4 conferring radiosensitivity.^191^ Crystal structures were reported for XRCC4(1–203) bound to Lig4 C-terminal BRCT domains (residues 654–911) (PDB 3II6),^192^ and for XRCC4(1–213) bound to Lig4's XRCC4 interaction region (XIR) (residues 755–782) (PDB: 1IK9),^193^ revealing the Lig4 N-terminus forms a coiled-coiled homodimer. The structures identified an extensive interface between XRCC4 and Lig4-BRCT, encompassing both Lig4-BRCT1 and BRCT2 domains and their connector region, which can be further explored for the design of inhibitors of this PPI. XRCC4-XLF is another PPI essential for the NHEJ's ligation complex whose disruption was shown to sensitize cells to IR.^194^ A 5.5 Å crystal structure of the complex XRRC4(1–157)–XLF(1–224) (PDB: 3Q4F) reveals a filamentous structure formed by the two proteins with a PPI interface mediated by the N-terminal regions of XRCC4 and XLF.^195^ A more recent 3.9 Å XRCC4(1–140)–XLF(1–224) (PDB: 3SR2) revealed that Leu115 in XLF, termed the Leu-lock, is a hot-spot of this PPI.^196^

***53BP1*** is a scaffold protein, which plays a central role in NHEJ initiation and HR suppression, whose PPIs hold promise as targets for NHEJ inhibitors.^197,198^ 53BP1 accumulates at chromatin near DSBs *via* interactions with modified histones, H2AK15-Ub and H4K20me2, through its focus formation region (FFR) and tandem Tudor domains, respectively.^197–200^ A small molecule that prevents 53BP1 accumulation at chromatin^201^ was rationally designed using crystal and an NMR structures of 53BP1 bound to a H4K20me2 peptide (PDB: 2IG0 and 2LVM).^200,202^ A fragment-like small molecule inhibitor, **UNC2170**, was designed to bind the lysine methyl binding cage of the 53BP1 Tudor domain formed by Tyr1502, Tyr1523, Phe1519, Trp149 (co-crystal structure, PDB: 4RG2) (Fig. 5A).^201^ This inhibitor attenuated class-switch recombination (CSR), but was not tested for its radiosensitization ability.

***Shieldin*** complex is a protector of DSB ends that promotes NHEJ and counters HR, providing another target to be considered for the NHEJ inhibitor design.^170–175^ Loss of shieldin proteins sensitizes DT40 and U2OS cells to radiomimetic agents, topoisomerase inhibitors, and IR.^203^ The structure of the shieldin complex comprised of Rev7, Shld1, Shld2 and Shld3 subunits is not yet available, however, a 3.45 Å crystal structure (PDB: 6KTO) has been reported for a Rev7 dimer bound to Shld2(1–64) and Shld3(1–52) fragments, which is a hub for the shieldin complex assembly.^204^ Rev7 dimer-breaking mutants attenuated NHEJ in CH12-F3 cells,^174^ suggesting disruption of Rev7 dimer with small molecules may attenuate NHEJ. In shieldin, the two Rev7 protomers interact with the two Shld3 Rev7-binding motifs (RBM, defined as Pxxx(A/P)P, where x is any residue^205,206^) that are also found in Rev7 interactors from other pathways.^204,207,208^ An inhibitor was identified that blocks the Rev7 PPI with an RBM motif from the Rev3 subunit of TLS DNA polymerase Polζ (reviewed below),^209^ suggesting that disruption of Rev7 PPIs with Shld3-RBMs might be a viable strategy for NHEJ inhibition.

### 6.3. HR inhibition

Inhibition of HR proteins can lead to radio- and genotoxic chemosensitivity,^169,210^ while synthetic lethal relationships of HR genes also exist that can be exploited for cancer treatment.^97,98^ HR has also been reported to facilitate resistance to alkylating therapy in glioblastoma cells more so than NHEJ.^211^ Together, this opens multiple avenues for targeting HR proteins for cancer therapy.

***MRN*** is a hexameric complex, composed of 2 copies of each MRE11, RAD50, and NBS1.^212,213^ MRE11 has DNA binding and nuclease activity, Rad50 hydrolyzes ATP to regulate nuclease activity, and NBS1 is a scaffold. The nuclease function of MRN promotes HR, however, MRN may also aid in NHEJ by tethering DSB ends.

Current inhibitors of MRN act against MRE11's nuclease activity.^214,215^ However, disruption of PPIs in the MRN complex could also provide a potential chemotherapeutic strategy. Mutations within PPI regions of NBS1, the scaffold protein of MRN, are correlated with cancer susceptibility,^216^ and knock down of NBS1 leads to IR sensitivity,^217^ alluding to the importance of NBS1 PPI for DSB repair. NBS1 contains an FHA domain, two tandem BRCT domains, a MRE11 interaction region (MIR, residues 474–531), and an ATM interaction motif mentioned above, all of which facilitate PPIs.^212,213^ For example, the structure of the N-terminal FHA and the two tandem BRCT domains of *S. pombe* NBS1 bound to a Thr-phosphorylated CtIP peptide (PDB: 3HUF) provides insights into MRN-CtIP recognition.^218^ Integral to the MRN complex is the MRE11 dimer stabilized by the MRE11-NBS1 PPI. The details of this PPI are available from a crystal structure of *S. pombe* MRE11/NBS1-MIR complex (PDB: 4FBQ).^219^ Furthermore, the human MRE11 core complex crystal structure (PDB: 3T1I) identified key residues in the MRE11 homodimerization interface.^220^ A crystal structure of the dimer zinc-hook domain from *P. furiosus* Rad50 (PDB: 1L8D) reveals that the highly conserved CXXC motif coordinates dimerization, whose mutations sensitize yeast cells to IR.^221^ The structures of *P. furiosus* Rad50 in complex with MRE11's Rad50 binding domain (PDB: 3QKR, 3QKS) were also determined providing details of this PPI.^222^

***Rad51*** is the ssDNA binding protein and recombinase responsible for strand invasion and homology searching in HR,^177,178^ which is often upregulated in radio- and chemo-resistant cancers.^8^ Several small molecule compounds were identified that inhibit Rad51–ssDNA binding, preventing the nucleoprotein filament formation (reviewed by Budke *et al.*^210^). In addition, inhibitors were designed to block Rad51 oligomerization, a process essential to form Rad51 filaments. Thus, the chemical inhibitor **RI-1** covalently binds C319 on the Rad51 oligomerization interface, reduces HR in cells, and increases cell death in response to genotoxic chemotherapy (Fig. 5B).^223^ An improved analogue **RI-2** was proposed, which circumvents high reactivity and low specificity of **RI-1** and reversibly inhibits Rad51 oligomerization. **RI-2** was also shown to sensitize cells to cross-linking agents.^224^

Another approach to disrupting Rad51 nucleoprotein filament formation is inhibition of the BRCA2-Rad51 PPI. BRCA2 loads Rad51 onto RPA-coated ssDNA, resulting in RPA displacement.^3–5,169,225^ BRCA2 harbors eight ∼35 amino-acid long BRC repeats featuring an FxxA motif in the region spanning residues 990–2100, with BRC repeats 1–4 binding to free Rad51 and BRC repeats 5–8 interacting with ssDNA-bound Rad51.^226^ Pellegrini *et al.*^227^ reported a crystal structure of the ATPase domain of Rad51 in complex with the BRC4 repeat of BRCA2 (PDB: 1N0W), revealing an extensive PPI interface that involves 28 residues of BRC4. A small molecule compound, **IBR2**, which has a phenylsulfonyl indolyl isoquinoline moiety, was identified as a disrupter of Rad51-BRCA2 PPI using a reverse Y2H-HTS assay and was validated to compete for Rad51 binding to BRC repeats by SPR (Fig. 5B).^228^ Docking studies suggest **IBR2** inhibits Rad51–BRCA2 PPI by blocking the hydrophobic pocket on Rad51 that accommodates the BRC FxxA motif. Furthermore, **IBR2** disrupts Rad51 oligomerization, induces its proteasomal degradation, attenuates HR, and induces apoptosis in cells. **IBR2** treatment in a chronic myeloid leukemia mouse model prolonged overall survival.^228^

Scott *et al.*^229^ used FBDD to identify two additional scaffolds, containing a tryptamine or a tryptophan ester moiety, that interrupt Rad51 association with the BRC FxxA motif by binding to Rad51's pocket described by Pellegrini *et al.*^227^ (Fig. 5B). The best fragments bind Rad51 with low μM range affinities and require further building and optimization to create drug leads.^229^ The same crystal structure^227^ was used by Cavalli and co-workers to conduct a virtual screen and identify four small molecule inhibitors of Rad51–BRCA2 PPI that bind to the FxxA pocket on Rad51.^230–232^ Two triazole-based compound hits identified in the screen were validated to disrupt Rad51–BRC4 PPI in an ELISA assay with EC_50_ of 53 and 25 μM, with the later working synergistically with PARPi to kill pancreatic cancer cells.^230^ Subsequent optimization led to a compound with EC_50_ of 8 μM which, however, did not induce synthetic lethality with PARPi.^231^ Going forth, the same group performed a virtual screen for another binding pocket on Rad51 that binds the BRCA2-BRC4 LFDE motif, which led to identification of dihydroquinoline pyrazoline derivatives as inhibitors of this PPI. Subsequent optimization to improve the Rad51–BRCA2 PPI disruption resulted in a compound **35d** (Fig. 5B), which exhibited an EC_50_ of 19 μM in the ELISA assay, had a binding affinity for Rad51 of 80 nM, inhibited HR by 54% in pancreatic cancer cells with a 20 μM concentration, and displayed synergy with PARPi in pancreatic cells with a concentration of 15 μM.^232^

### 6.4. Alt-NHEJ and SSA inhibition

***Alt-NHEJ***, also termed Polθ-mediated end joining,^179–181^ has recently garnered popularity as a potential chemotherapeutic target.^181^ Polθ is synthetic lethal with ATM and various HR genes. Deficiency in Polθ causes sensitivity to ionizing radiation, DSB inducing drugs, and PARP inhibitors. Furthermore, Polθ overexpression is correlated with poor prognosis in cancer patients.^179^ Polθ has Rad51 binding motifs and is reported to bind to Rad51 in immunoprecipitation assays. It is not yet established if this PPI is necessary for alt-NHEJ function and therefore more investigation is needed.^180^**SSA** is another pathway of chemotheraputic interest,^166,167,182^ as Rad52 enhances the synthetic lethality of PARPi and BRCA deficiency.^233,234^ Thus, inhibitors of Rad52 were identified using fluorescence-quenching HTS assay for Rad52's ssDNA annealing activity, which selectively kill BRCA deficient cells.^235^

## 7. Targeting NER, BER and MMR

More frequent than DSBs, which is the most catastrophic DNA damage, are less severe DNA lesions such as chemically altered nucleotides or mispaired bases counteracted by several major DNA repair mechanisms.^3–5^ Thus, intra-strand cross links, pyrimidine dimers and bulky adducts are primarily repaired by nucleotide excision repair (NER),^3–5,236–238^ small base modifications are mitigated by base excision repair (BER),^3–5,239–241^ and nucleotide mismatches are corrected by mismatch repair (MMR).^3–5,242–244^ In addition, a combination of NER, HR and TLS (discussed below) is used to unhook and repair inter-strand cross-links (ICL) through a complex Fanconi Anemia (FA) pathway.^245,246^ In this section, we briefly overview the three major DNA repair pathways and discuss their targeting with small molecule PPI inhibitors.

### 7.1. NER, BER and MMR pathways

**Fig. 6:**
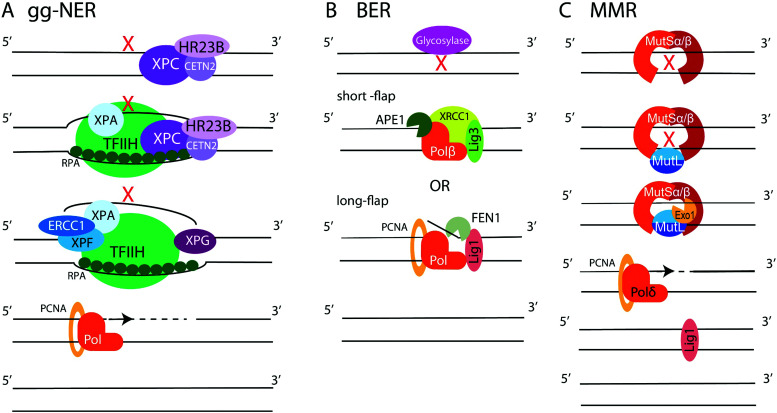
Schematic of NER, BER, and MMR. (A) gg-NER begins with recognition of a helix-distorting lesion by XPC/HR23B/CETN2, which recruits TFIIH to unwind DNA around the lesion. RPA binds the undamaged ssDNA strand, while XPA/XPF/ERCC1 recognizes the damage and, along with XPG associated with TFIIH, cuts DNA fragment around the lesion. A polymerase fills the gap and XRCC1-Lig3 ligates the newly synthesized DNA. (B) BER. One of 11 DNA glycosylases recognizes DNA damage and excises modified base, creating abasic site. In the short-patch BER, APE1 nicks DNA next to abasic site, and Polβ fills a single nucleotide gap and removes abasic sugar by its dRP lyase activity. In the long-parch BER. Polβ or Polδ/ε synthetizes longer DNA stretch, creating a flap removed by FEN1. The final nick is sealed by XRCC1-Lig3 or Lig1. (C) MMR. A mismatch or a deletion-insertion loop is recognized by MutSα or MutSβ. MutL binds MutS and recruits Exo1, which excises the mismatch. The gap is filled by Polδ and nick ligated by Lig1.

***NER*** is employed to remove bulky lesions that distort the DNA helix and includes two sub-pathways, the global genome NER (gg-NER) and the transcription coupled NER (TC-NER).^3–5,236–238^ The first step of the gg-NER is recognition of DNA damage by the Xeroderma Pigmentosum complementation group C protein (XPC) in complex with HR23B (Rad23B) and Centrin2 (CETN2) (Fig. 6A). In addition, UV-induced cyclobutane pyrimidine dimers (CPDs) can be recognized by ultraviolet radiation-DNA damage binding protein 1 and 2 (DDB1 and DDB2), which then recruit XPC. A ten-subunit complex, the transcription initiation factor IIH (TFIIH), recognizes XPC bound to DNA and unwinds the DNA region around the lesion, exposing ssDNA. RPA binds the undamaged DNA strand, while xeroderma pigmentosum group A protein (XPA) binds chemically alerted nucleotides and recruits the endonuclease complex, XPF-ERCC1. The XPF-ERCC1 complex and XPG nick the DNA on the 5′ and 3′ end of the lesion, respectively, creating an exposed ssDNA gap within the double helix. DNA polymerases (such as Polδ, Polε, or Polκ) with the aid of PCNA and replication factor C (RFC) carry out DNA replication to fill the gap, and Lig1 or XRCC1-Lig3 ligases seal the final nick. The second NER sub-pathway, TC-NER, is initiated by stalling of the RNA polymerase II (PolII) at a bulky DNA lesion. Cockayne syndrome protein B (CSB) binds to the stalled RNA polymerase and recruits cockayne syndrome protein A (CSA) which, in turn, recruits additional factors to remodel chromatin, allowing backtracking of the stalled RNA polymerase II followed by recruitment of TFIIH. Then, the NER pathway commences as described above.

***BER*** repairs single-base DNA damage that has not resulted in significant distortion of the DNA helix. At least 11 DNA glycosylases in human cells detect and excise modified and flipped-out DNA bases, creating abasic sites and initiating either short- or long-patch BER (Fig. 6B).^3–5,239–241^ In short-patch repair, the AP endonuclease (APE1) nicks the backbone 5′ to the abasic site and recruits DNA polymerase Polβ to fill a single nucleotide gap and remove abasic sugar *via* its deoxyribose phosphate (dRP) lyase activity. This is followed by sealing of the final nick by XRCC1-Lig3 or Lig1 ligases. In long-patch repair, Polβ or a replicative polymerase Polδ or Polε (during S or G2 phases) synthetizes 2–10 nucleotide DNA fragment to fill the gap, displacing several nucleotides from the opposite strand. The generated ssDNA flap is removed by flap endonuclease 1 (FEN1), followed by ligation of the final nick by Lig1. Additionally, ssDNA break repair (**SSBR**) is performed in a manner similar to BER.^100,101,241^ First, the SSB is detected by PARP1. Then, APE1, polynucleotide kinase/phosphatase (PNKP), aprataxin, and tyrosyl-DNA phosphodiesterase 1 (TDP1) modify the DNA break ends. Lastly, the exposed ssDNA is filled in and ligated using the BER protein complexes.

***MMR*** specializes on the repair of base-pair mismatches and loops caused by insertions-deletions (Fig. 6C).^3–5,242–244^ The heterodimer MSH2/MSH6 (MutSα) scans DNA for mis-incorporated bases, while the MSH2/MSH3 (MutSβ) heterodimer seeks out insertion-deletion loops. When MutSα or MutSβ detects a mismatch, another heterodimer, MLH1/PMS2 (MutLα), is recruited to coordinate repair. MutLα aided by PCNA and RFC recruits the endonuclease Exo1, which carries out mismatch excision creating an RPA-coated ssDNA gap. The gap is filled by Polδ, and Lig1 performs the final ligation step. Mutations in MMR proteins are implicated in Lynch syndrome, a predisposition to colon cancer.^247^

### 7.2. Targeting NER PPIs

NER is involved in repairing DNA damage caused by genotoxic chemotherapies, particularly front-line platinating and alkylating agents, and therefore provides a promising target for cancer sensitization to these drugs.^102–106,237^ Multiple PPIs are essential to the NER pathway that could potentially be inhibited for chemotherapeutic development.

**Fig. 7:**
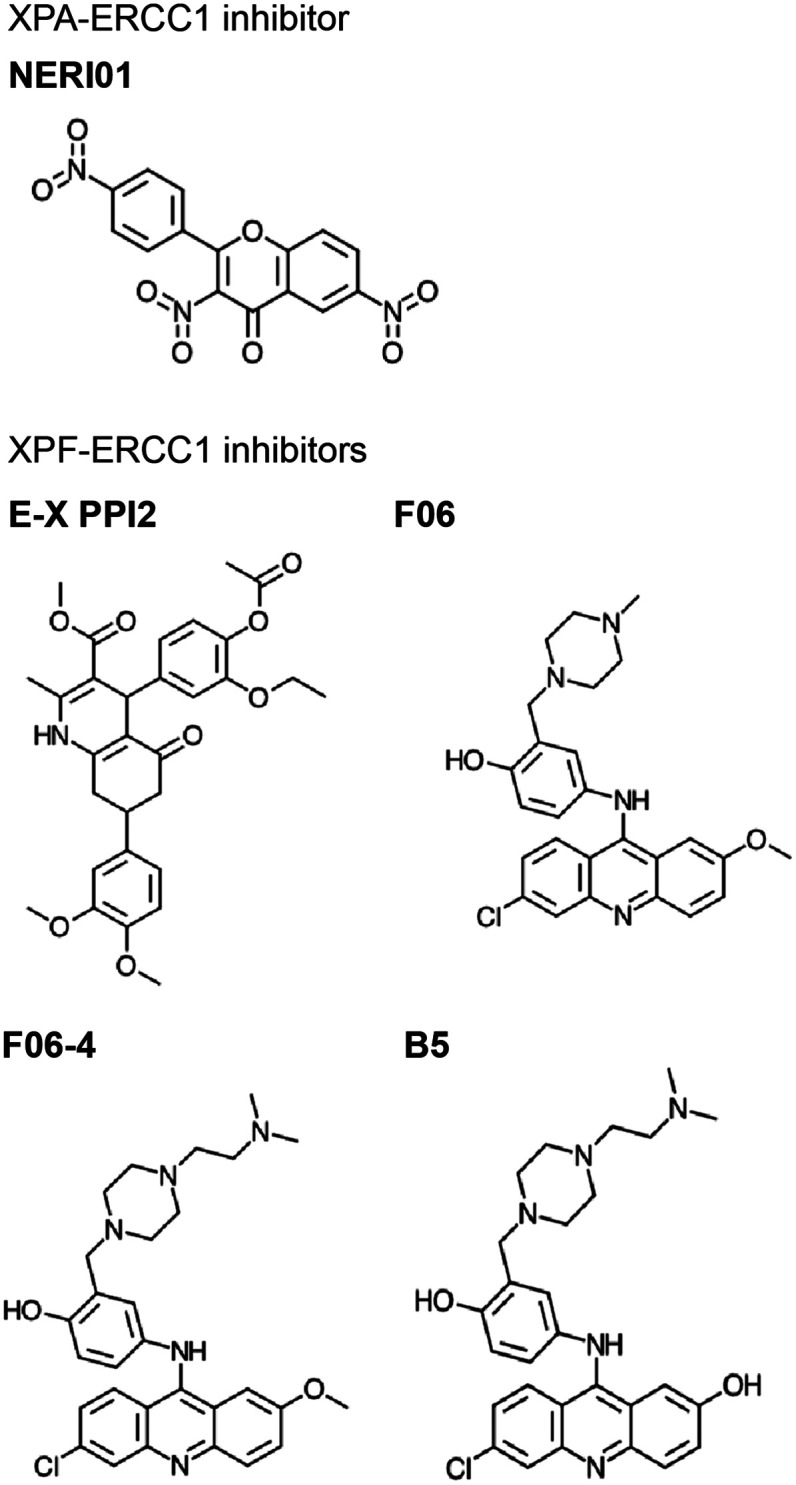
Inhibitors of NER PPIs that target XPA-ERCC1 (top) and XPF-ERCC1 (bottom) complex formation.

***XPA*** is a major NER scaffold protein with an extensive network of binding partners, including DNA, RPA, DDB1/2, TFIIH, ERCC1, PCNA, and ATR (reviewed by Sugitani *et al.*^248^). Mutation to the XPA gene causes Xeroderma pigmentosum (XP), a syndrome of light sensitivity and a high risk of cancer, suggesting XPA is crucial to the NER pathway.^249^ XPA binds ERCC1 to initiate the 5′ incision.^250^ Mutations in XPA that disrupt the XPA-ERCC1 PPI limit NER activity,^251^ and decreased expression of either protein leads to UV and chemotherapeutic sensitivity.^252^ Solution NMR structure is available of the ERCC1 complex in complex with an XPA peptide, permitting a SBDD for this PPI (PDB: 2JNW).^253^ Researchers at the University of Alberta successfully conducted SBVS of a 50 000 small molecule library against 10 ERCC1 models to identify inhibitors of the XPA-ERCC1 PPI, followed by docking and MD simulations to narrow down the hits.^254^ One of the identified inhibitors, **NERI01**, is predicted to facilitate six hydrogen bonds with ERCC1. **NERI01** was validated to bind ERCC1 in fluorescent quenching assay and sensitized colon cancer cells to UV radiation (Fig. 7).

***XPF-ERCC1*** is another complex necessary for the 5′ incision in NER, which also participates in HR and NHEJ.^255^ XPF-ERCC1 is a major chemotherapeutic target with a few inhibitors identified.^256–260^ XPF and ERCC1 form a heterodimer *via* mainly hydrophobic interactions between their double helix–hairpin–helix (HhH_2_) C-terminal regions (PDB: 2A1J, 1Z00, 6SXA, 6SXB).^261–263^ Researchers at the University of Edinburgh utilized *in silico* SBVS to target pockets on the XPF binding site for ERCC1, resulting in identification of 29 compound hits, 4 of which were confirmed to bind XPF by SPR.^256^ The most potent hit, **E-X PPI2**, impeded NER in cells with an IC_50_ of 20 μM (Fig. 7). **E-X PPI2** also sensitized melanoma cells to cisplatin and disrupted XPF-ERCC1 dimerization in ovarian cancer cells. In another study, researchers at the University of Lyon performed *in silico* SBVS and identified 73 hits predicted to bind XPF's ERCC1 interface.^257^ SBVS hits were validated *via* methylthiazoletetrazolium (MTT) cytotoxicity assays to test for synergy with cisplatin, mitomycin C (MMC), and UVC irradiation. Compounds that exhibited synergy were then tested for their ability to bind to XPF's C-terminus by SPR and fluorescence quenching experiments. In these assays, compound **F06** was the strongest binder, and also disrupted the XPF-ERCC1 PPI in cells (Fig. 7). However, the authors raised concerns about potency and safety, admitting its further optimization is needed to be considered a drug lead. In the follow-up study, optimization of **F06** was carried out *via* docking, pharmacophore modeling, and MD, resulting in seven **F06** derivatives.^258^ One of the analogues, **F06-4** (Fig. 7), sensitized colon cancer cells to UV radiation and cyclophosphamide, exhibited IC_50_ of 0.33 μM (improved 5-fold over 1.86 μM for **F06**), and displayed favorable physicochemical and ligand efficiency profiles, suggesting it may be effective *in vivo*.^259^ A second round of **F06** optimization culminated in the design of **B5**, which inhibited nuclease activity of the XPF-ERCC1 complex with an IC_50_ of 0.49 μM (Fig. 7).^258^ Taken together, these studies indicate that further optimization of the **F06** scaffold is a promising avenue to develop effective chemotherapeutic sensitizers that inhibit XPF-ERCC1 PPI in NER.

***TFIIH*** is a protein complex made of ten subunits.^237,264^ A 4.4 Å resolution Cryo-EM structure of TFIIH was reported (PDB: 5OF4), revealing overall architecture of the complex.^265^ In addition to its central role in NER, TFIIH also functions as a transcription initiator, so targeting this complex should be executed in a manner specific to NER to avoid toxicity. The N-terminal pleckstrin homology (PH) domain of the p62 subunit of TFIIH binds and recruits the 3′ nuclease, XPG. Truncation of this domain reduces incision and excision during NER, but does not affect transcription, suggesting this PPI is specific to NER.^266^ Solution NMR structure of the p62-XPG yeast homolog, Tfb1-Rad2 complex, is available (PDB: 2LOX).^267^ Although the human p62-XPG PPI has not been structurally characterized, apo and DNA-bound structures of XPG are available (PDB: 6TUW, 6TUX),^268^ as well as solution NMR structures of the p62 PH domain (PDB: 1PFJ),^266,269^ potentially allowing a SBDD of inhibitors of the p62-XPG PPI.

### 7.3. Targeting BER PPIs

BER is employed to remove several types of lesions induced by genotoxic chemotherapy, especially alkylation therapy,^3–5,239–241^ and mitigate oxidative DNA damage elevated in the highly oxidative tumor environment.^270,271^ Inhibition of BER DNA polymerase Polβ is synthetic lethal in cancers that are deficient in MMR genes,^110^ while XRCC1 deficiency is synthetic lethal with PARP and causes hypersensitivity to genotoxic chemotherapy.^272^ Overexpression of BER components are correlated with poor survival in gastric cancer.^273^ Together, these data highlight potential benefits of BER inhibition by targeting key PPIs of this pathway for treatment of multiple forms of cancer.

***XRCC1*** is a BER scaffold central to the assembly of enzymatic complexes in BER and SSBR, which interacts with DNA glycosylases, APE1, Polβ, DNA ligases, and PARP1.^239–241,274^ XRCC1 has three domains, the N-terminal domain (X1NTD) that binds polβ, the central BRCT domain (X1BRCTa) that binds PARP1, and the C-terminal BRCT domain (X1BRCTb) that binds Lig3.

The XRCC1–Polβ PPI is important for cell resistance to alkylating agents.^275^ Although Polβ catalytic inhibitors are available, they are only moderately efficient and little data suggests they are specific,^276,277^ indicating that PPI inhibition may be a viable alternative. X-ray crystal structures were solved for both reduced and oxidized X1NTD in complex with the Polβ catalytic domain (PDB: 3K75, 3LQC).^278^ NMR chemical shift mapping of the X1NTD–Polβ interface is also available, which deviates from that observed by crystallography.^279^ The crystal structure revealed an extensive X1NTD–Polβ PPI interface involving 40 residues of Polβ. The PPI is strengthened by oxidation that stimulates a disulfide bond formation between C2–C12 of X1NTD, which is thought to be a molecular switch that increases BER activity during oxidative stress.^278^ Mutational analysis of the PPI interface identified potential hot-spot residues in X1NTD that can be targeted with small molecules.^280^ These studies provided plentiful structural data for SBDD of XRCC1–Polβ PPI inhibitors.

The XRCC1–Lig3 complex, which is utilized for ligation in both BER and NER, is another attractive PPI for targeting with small molecule inhibitors. XRCC1 and Lig3 interact through their C-terminal BRCT domains, with X-ray crystal structures available for the BRCT-mediated heterodimer (PDB: 3PC8, 3QVG, 6WH1), and for BRCT domain homodimers of both proteins (PDB: 3PC6, 3PC7, 6WH2).^281,282^ The dimerization interface is similar in hetero- and homo-dimers, suggesting the proteins are competitive binding partners. The available structural data primes XRCC1–Lig3 for possible SBDD of small molecule PPI inhibitors.

XRCC1-APE1 is another BER PPI of pharmaceutical interest. APE1 inhibition was proposed as a strategy to potentiate cytotoxicity of alkylating agents.^283^ The APE1 binding region of XRCC1 was mapped to the BRCTa domain and a hinge connecting NTD and BRCTa.^284,285^ Although the crystal structures of APE1 are available (PDB: 1DE8, 1DE9, 1DEW),^286^ the structure of XRCC1–APE1 complex is yet to be determined.

### 7.4. Targeting MMR

Few reports suggest that targeting MMR can provide a viable chemotherapeutic strategy, as loss of MMR is mutagenic and can lead to an oncogenic transformation.^12,287,288^ Although some studies report that loss of MMR can sensitize cells to DSB-inducing agents,^289^ other reports suggest that loss of MMR can cause resistance.^290^ MutSα (MSH2/MSH6 heterodimer) expression promotes sensitivity to cisplatin treatment, while its inhibition would lead to cisplatin resistance.^291^ Loss of MMR also causes resistance to alkylating therapy.^292^ Intriguingly, MMR deficiency is associated with a robust response to immunotherapy; however, current hypotheses predict this is due to microsatellite instability that has accumulated over time, rather than a direct consequence of MMR deficiency.^293^ Although plenty of PPIs are imperative to this pathway, there is little rationale that their inhibition has non-toxic chemotherapeutic potential.

## 8. Targeting TLS

### 8.1. TLS mechanisms

**Fig. 8:**
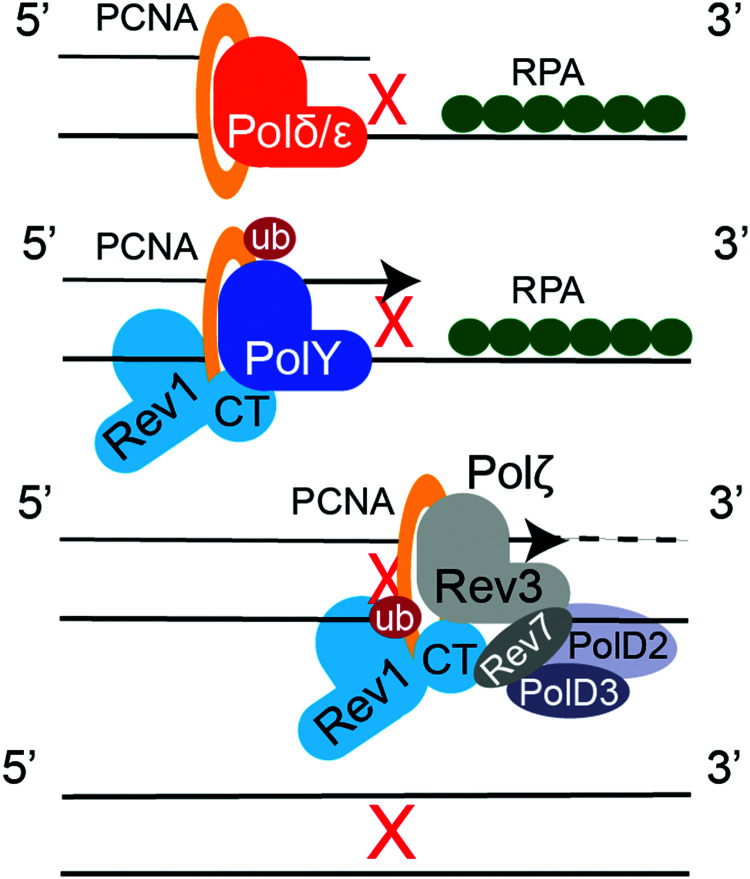
Two step Rev1/Polζ-dependent TLS. After PCNA monoubiquitination by Rad6/Rad18, an inserter Y-family TLS polymerase replaces a replicative polymerase, Polδ or Polε, and inserts nucleotides across the DNA lesion. The extender TLS polymerase Polζ continues replication past the site of DNA damage.

Translesion synthesis (TLS) is a mechanism of cellular DNA damage tolerance (DDT) that allows bypass replication over sites of DNA damage without the need of immediate repair.^16–19^ When a replicative polymerase, Polδ or Polε, stalls at a DNA lesion, Rad6/Rad18-dependent mono-ubiquitination of PCNA at K164 signals recruitment of TLS DNA polymerases, which take over replication and synthetize a stretch of DNA across from the lesion (Fig. 8).^294,295^ TLS DNA polymerases also participate in post-replication gap filling, which is most prevalent during G2/M.^296–298^ Some DNA lesions can be bypassed by a single TLS DNA polymerase, as exemplified by the accurate and efficient replication over TT-CPDs by Polη.^299,300^ However, replicative bypass of most DNA lesions occurs by a two-step Rev1/Polζ-dependent TLS,^301,302^ which involves recruitment of multiple TLS DNA polymerases through multivalent PPIs with PCNA and Rev1.^19^ Y-family TLS polymerases bind PCNA trough their PCNA-interacting protein box (PIP-box) motifs (Polη, Polι, and Polκ),^303^ or a BRCT domain (Rev1).^304,305^ These PPIs are enhanced by ubiquitin binding domains of TLS enzymes (UBM in Polι/Rev1, UBZ in Polη/Polκ) that interact with ubiquitin moiety on ub-PCNA.^306,307^ The multi-protein TLS complex is additionally stabilized by the Rev1 C-terminal domain (Rev1-CT), a critical TLS scaffold that binds the Rev7 subunit of TLS polymerase Polζ (Rev3/Rev7/PolD2/PolD3 complex^308,309^) and Rev1-interacting regions (RIRs) of Polη, Polι, Polκ, and PolD3.^310–316^ In the first step of Rev1/Polζ-dependent TLS, an inserter Y-family polymerase, Polη, Polι, Polκ or Rev1, inserts a nucleotide across the lesion. In the second step, an extender polymerase, often Polζ, continues DNA replication past the lesion-distorted DNA primer–template (Fig. 8).

### 8.2. Inhibitors of TLS PPIs

TLS polymerases are implicated in the replicative bypass of DNA adducts formed by genotoxic platinating and alkylating agents, increasing cancer cell survival after first-line chemotherapy.^113–116^ Furthermore, TLS polymerases are extremely error-prone, and induce mutagenesis that allows cancer cells to adapt and develop drug resistance.^113,114^ TLS inhibitors may potentially serve as combination drugs to enhance efficacy of first-line genotoxic therapy and reduce mutagenesis, delaying the onset of chemoresistance.^13,117,118^ Catalytic inhibition of TLS DNA polymerases is difficult due to structural similarity of active sites. A few non-selective TLS polymerase catalytic inhibitors have been identified that also bind to replicative polymerases.^317–319^ Therefore, PPI disrupters may be the preferred route for TLS inhibition.

**Fig. 9:**
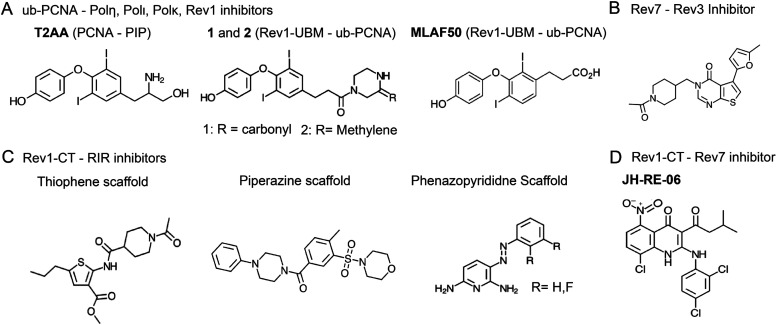
Inhibitors of TLS PPIs between (A) Y-family TLS polymerases and ub-PCNA, (B) Rev7 and Rev3-RBM, (C) Rev1-CT and RIR motifs, and (D) Rev1-CT and Rev7.

***Ub-PCNA PPIs*** that recruit TLS polymerases to sites of DNA damage are critical to TLS function. Researches at St. Jude's Children's Hospital designed an inhibitor, T2 amino alcohol (**T2AA**), that disrupts PCNA PPIs with the PIP-box motifs of replicative DNA polymerases and other replication-related proteins (*e.g.* PDB: 1AXC^320^), and inhibits DNA replication (Fig. 9A).^321^ This compound also disrupted PCNA-Polη PPI *in vitro* and localization of TLS polymerases to PCNA in cells, suggesting it acts to attenuate TLS.^322^ In another study from this research group, two structurally related inhibitors, **1** and **2**, were identified in an AlphaScreen^323^ that bind to Rev1-UBM2 and inhibit Rev1 PPI with ub-PCNA (Fig. 9A).^324^ Binding of the compounds to Rev1–UBM2 was confirmed by STD-NMR and protein-based ^1^H–^15^N HSQC NMR experiments. Compound **1** restricted Rev1 recruitment, limited ICL repair, and sensitized U2OS cells to cisplatin. The same research group also identified a small molecule, **MLAF50**, as a binding partner of Rev1–UBM2 and disrupter of its PPI with ub-PCNA (Fig. 9A), confirmed that the compound binds Rev1–UBM2 and is displaced from the complex by ubiquitin using NMR, and demonstrated that **MLAF50** displaces Rev1 from chromatin in U20S cells.^325^

***Rev7-Rev3 PPI*** is essential for assembly and function of the ‘extender’ B-family TLS polymerase Polζ.^19^ Rev7 is an accessory subunit of Polζ that forms a homodimer interacting with the two Rev7-binding motifs (RBM1 and RBM2; consensus sequence Pxxx(A/P)P^205,206^) of Polζ's Rev3 catalytic subunit. X-ray crystal structures are available for a dimer-breaking R124A Rev7 mutant bound to Rev3-RBM1 and RBM2 peptides (PDB: 3ABD, 3ABE, 6BC8), revealing a binding mechanism in which a ‘safety-belt’ loop of Rev7 wraps around the RBM peptide.^205,326^ The above research group at St. Jude's Children's Hospital performed a HTS AlphaScreen assay^323^ and identified a compound with a furan ring moiety capable of disrupting Rev7 PPI with the Rev3-RBM1 peptide.^209^ An optimized analog of this compound (Fig. 9B) was shown to bind Rev7 by NMR, and sensitized HeLa cells cisplatin. It should be noted that Rev7 also interacts with proteins from several other pathways, including Shld3 (see section 6.2 on NHEJ inhibition), chromosome associated maintenance protein (CAMP), and Ras-associated nuclear protein (RAN), in a similar manner to Rev3,^204,207,208^ suggesting that the Rev7/RBM PPI inhibitors may have a complex mechanism of action.

***Rev1-CT PPIs*** with RIR motifs of Polη, Polι, Polκ, and PolD3 (a Polζ subunit), and with the Rev7 subunit of Polζ are the scaffolding interactions necessary for assembly and function of the multi-protein TLS complex.^19^ RIR motifs were also identified in proteins from other pathways, including the BER scaffold XRCC1.^327^ Rev1-CT is a four-helix bundle that has two independent binding interfaces for TLS DNA polymerases, the N-terminal interface for RIR motifs and the C-terminal interface for Rev7.^311–315^ Several X-ray crystal and solution NMR structures are available for apo Rev1-CT and its complexes with Polη, Polκ and PolD3 RIR motifs (PDB: 2LSJ, 2LSG, 2LSK, 2LSY, 2N1G, 4FJO),^311–314^ as well as a crystal structure of the triple Rev1-CT/Rev7/Rev3-RBM1 complex (PDB: 3VU7),^316^ revealing details of Rev1-CT PPIs.

RIR motifs (nFFhhhh, where n is a N-capping residue, h is a helix-forming residue^310,311^), upon binding, form an α-helix that inserts side chains of FF residues into a pocket on Rev1-CT,^311–314^ which provides a druggable ‘hot-spot’ for the development of PPI inhibitors. In a series of works, researchers at the University of Connecticut identified multiple small molecule TLS inhibitors targeting Rev1-CT/RIR PPIs.^328–332^ Two initial scaffolds, thiophene and piperazine, identified in an FP-based HTS assay disrupted the Rev1-CT PPI with a fluorescently tagged FAM-Polκ-RIR peptide (Fig. 9C).^328^ The thiophene scaffold compound was shown to bind the RIR-interface of Rev1-CT by NMR, sensitized fibrosarcoma cells to cisplatin, and reduced cisplatin-induced mutagenesis. An extended HTS screen using 10 000 compounds from the ChemBridge DIVERSet library identified multiple Rev1-CT/RIR inhibitors, which were grouped into five clusters based on structural similarity with the two clusters corresponding to the initial thiophene and piperazine scaffolds.^329^ The following studies reported a SBVS, which identified several new chemotypes that disrupt Rev1-CT/RIR PPI,^330^ and a SBDD utilizing structural knowledge of the Rev1-CT/RIR interface, which identified a phenazopyridine scaffold mimicking the RIR FF pair as an inhibitor of this PPI (Fig. 9C).^331^ An extensive experimental validation of the identified inhibitors was performed, including FP- or FI-based displacement assays, protein-based and ^19^F ligand-based NMR binding studies, and cellular assays showing that several scaffolds enhance cisplatin sensitivity of cultured cells. Recently, a 2.5 Å resolution X-ray crystal structure has been determined for the most potent phenazopyridine compound bound to Rev1-CT (in the context of Rev1-CT/Rev7/Rev3-RBM1 complex; PDB: 6WS5), which guided the design of second-generation PAP derivatives exhibiting low μM binding affinities to Rev1-CT that are improved by an order of magnitude relative to the first-generation compounds.^332^

The second PPI interface of Rev1-CT binds the Rev7 subunit of Polζ, an interaction integral to the Polζ recruitment to DNA.^315,316^ Researchers at Duke University and Massachusetts Institute of Technology utilized an ELISA assay HTS and identified, a 1,4-dihydroquinolin-4-one derivative, **JH-RE-06**, as a disruptor of Rev7/Rev1-CT PPI (Fig. 9D).^333,334^**JH-RE-06** binds Rev1-CT with a *K*_d_ of 0.42 μM in ITC assays, and disrupts Rev1-CT/Rev7 PPI with an IC_50_ of 0.78 μM in an AlphaScreen assay.^323^ A 1.50 Å crystal structure of the inhibitor bound to Rev1-CT (PDB: 6C8C) revealed a unique mechanism of PPI inhibition, in which **JH-RE-06** binds the Rev7 interaction site on Rev1-CT and induces dimerization of Rev1-CT enclosing the inhibitor within the dimer interface.^333^ The inhibitor increased cytotoxicity and reduce mutagenesis in cells treated with cisplatin, and also improved cisplatin efficacy in a mouse model, halting tumor growth with the combination treatment of **JH-RE-06** and cisplatin.^333^ In a subsequent work, the authors demonstrated that **JH-RE-06** enhances tumor response to chemotherapy by inducing senescence.^334^

***Additional TLS PPIs*** that may be targeted with small molecules and their implications in cisplatin resistance have been extensively studied. For example, a triple Rev7 mutant, K44A,R124A,A135D, that abrogates Rev7 dimerization was unable to induce cisplatin resistance in Rev7 knockout cells, indicating the Rev7 dimerization interface is a possible target for chemotherapeutic intervention.^326^ In addition to Rev3 and Rev7, Polζ contains two accessory subunits, PolD2 and PolD3, that form a heterodimer interacting with a Fe–S cluster in the Rev3 C-terminus.^308,309^ PolD2 and PolD3 that are also subunits of the replicative DNA polymerase, Polδ, mediate PPIs and have no catalytic activity. Mutation of Rev3's Fe–S cluster that interacts with PolD2 decreases mutagenesis, suggesting disruption of this PPI may be sufficient to attenuate TLS.

Rad6–Rad18 complex responsible for PCNA ubiquitination is important, but not essential for TLS.^335^ Overexpression of Rad6 ubiquitin conjugating enzyme is correlated with cisplatin resistance in triple-negative breast cancer.^336^ Therefore, Rad6–Rad18 system may provide another possible target for chemotherapeutic intervention. Catalytic inhibitors of Rad6 have been described that reduce Rad6-ubiquitin thioester formation.^337,338^ Potential targets for the Rad6–Rad18 PPI inhibitors may include interfaces of Rad6 with the Rad18 N-terminal ring domain, and with the Rad18 C-terminal Rad6-binding domain (PDB: 2YBF).^339^

## 9. Conclusions

Attacking cancer's aberrant DDR with small molecule inhibitors is a promising route to creating new chemotherapies, as exemplified by the development of PARP inhibitors that received clinical approval for treatment of BRCA deficient cancers.^9^ To date, most inhibitors have been designed against traditionally “druggable” proteins with the majority targeting the enzyme active sites. This greatly restricts the number and quality of drug targets and often results in drugs with poor selectivity. The DDR signaling network provides a vast number of protein targets that mediate various crucial PPIs, which can be targeted with small molecules with high efficiency and specificity. Genes encoding these proteins are essential for cancer cell survival and often exhibit synthetic lethal relationships with other DDR genes, providing abundant opportunities for the development of new drugs that selectively kill cancer cells and/or sensitize cancers to existing therapies. The examples discussed in this review highlight the immense potential of targeting DDR PPIs for the development of novel cancer chemotherapies. The discussion, however, covered only a few essential PPI targets in several major DDR pathways, providing a sparce overview of numerous therapeutic opportunities within a vast DDR PPI network. The detailed picture of this network is only now beginning to emerge, suggesting multiple new PPIs targets for anti-cancer drug development will be identified in the future.

## Conflicts of interest

There are no conflicts to declare.

## Supplementary Material
